# Asymmetric synthesis of host-directed inhibitors of myxoviruses

**DOI:** 10.3762/bjoc.9.23

**Published:** 2013-01-30

**Authors:** Terry W Moore, Kasinath Sana, Dan Yan, Pahk Thepchatri, John M Ndungu, Manohar T Saindane, Mark A Lockwood, Michael G Natchus, Dennis C Liotta, Richard K Plemper, James P Snyder, Aiming Sun

**Affiliations:** 1 Emory Institute for Drug Development, Emory University, 1515 Dickey Drive, Atlanta, GA 30322, USA; 2 Department of Pediatrics, Emory University School of Medicine, 2015 Uppergate Drive, Atlanta, GA 30322, USA; 3 Children’s Healthcare of Atlanta, 2015 Uppergate Drive, Atlanta, GA 30322, USA; 4 Department of Chemistry, Emory University, 1515 Dickey Drive, Atlanta, GA 30322, USA; 5 Department of Microbiology & Immunology, Emory University School of Medicine, 1510 Clifton Road, Atlanta, GA 30322, USA

**Keywords:** asymmetric synthesis, benzimidazole, host-directed, myxovirus, small molecule inhibitor

## Abstract

High-throughput screening (HTS) previously identified benzimidazole **1** (JMN3-003) as a compound with broad antiviral activity against different influenza viruses and paramyxovirus strains. In pursuit of a lead compound from this series for development, we sought to increase both the potency and the aqueous solubility of **1**. Lead optimization has achieved compounds with potent antiviral activity against a panel of myxovirus family members (EC_50_ values in the low nanomolar range) and much improved aqueous solubilities relative to that of **1**. Additionally, we have devised a robust synthetic strategy for preparing **1** and congeners in an enantio-enriched fashion, which has allowed us to demonstrate that the (*S*)-enantiomers are generally 7- to 110-fold more potent than the corresponding (*R*)-isomers.

## Introduction

Myxoviruses are divided into two evolutionarily distinct yet related families: the orthomyxoviridae, which is composed largely of the influenza viruses, and the paramyxoviridae, which includes respiratory syncytial virus (RSV), measles virus (MeV), human parainfluenza virus (HPIV) and others [1]. Because myxoviruses are responsible for the majority of human morbidity and mortality cases due to viral respiratory illness globally, a therapeutic strategy that targets these viruses could have a substantial impact on human health [2–5]. Although antivirals typically seek to disable viral proteins, cellular host proteins can also be targeted, as is the case with selzentry, which inactivates the coreceptor (CCR5) for human immunodeficiency virus (HIV) entry [6]. The former approach is likely to yield compounds with a narrow spectrum of antiviral activities, and such inhibitors will certainly face the inevitable challenge of resistance [7–8]. Our research group has been actively engaged in the identification of small molecule inhibitors against myxoviruses in recent years [8–12], with a particular focus on the development of agents that target host-cell proteins enabling viral reproduction. Advantages of this strategy include a vastly expanded list of potential targets; a broader spectrum of activity, because many of the relevant host proteins are shared among related viruses; and, in principle, less susceptibility to the development of resistance.

**Figure 1:**
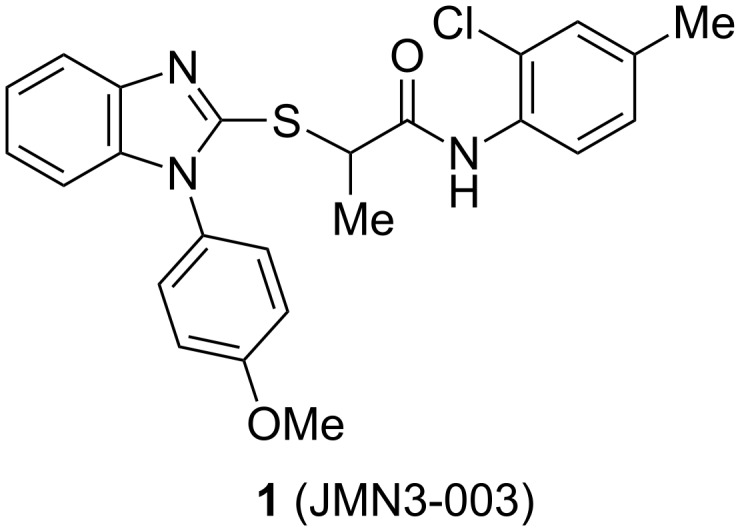
Structure of first-generation lead compound **1**.

Using high-throughput screening, in combination with counter-screening for detecting a broadened viral target spectrum that extends to other pathogens of the myxovirus families, our research group has been successful in identifying small-molecule antiviral hits resident in host cells [13]. One such molecule recently described is benzimidazole **1** [14] (Figure 1). Although the compound is active in vitro against a number of different para- and orthomyxoviruses, **1** has poor water solubility (<15 μg/mL), which may contribute to its low oral bioavailability [15]. Additionally, it was shown that the methyl group at the stereogenic center alpha to the carbonyl is important for biological activity [8,12,14]. Compound **1** was previously prepared as the racemate, but subsequently separated into enantiomers by chiral HPLC. To enable large-scale preparation of the pure isomers for further pharmacokinetic and animal studies, we present here an asymmetric synthesis of **1** and its congeners with improved aqueous solubility and antiviral potency.

## Results and Discussion

**Design.** We previously reported a series of compounds with antiviral activity against a number of myxoviruses [8,12,14]. Structure–activity relationship (SAR) studies suggested both the 2-chloro-4-methylanilide and the central α-thiopropionamide to be moieties that confer good activity. Relatively unexplored in our previous work was the importance of the *p*-methoxyphenyl ring as well as the influence of the stereochemistry at the chiral center. In the current work, we examine the replacement of the *p*-methoxyphenyl ring with basic moieties that may increase aqueous solubility while maintaining activity, and we also developed synthetic routes to produce each enantiomer of these compounds.

**Scheme 1:**
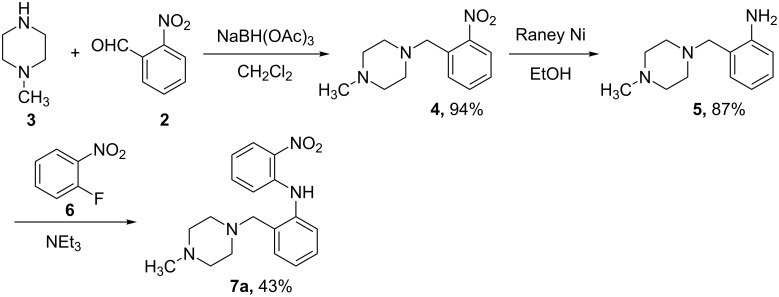
Synthesis of anilino nitrobenzene **7a**.

**Scheme 2:**
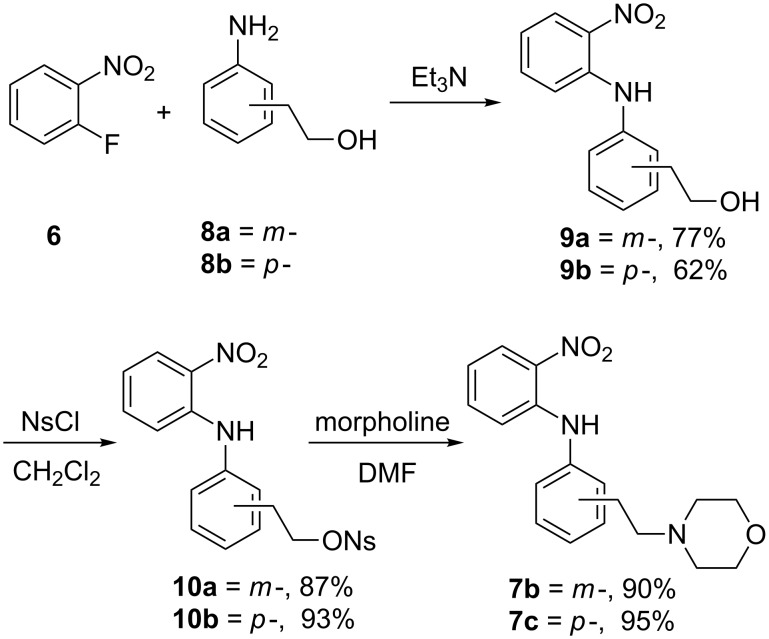
Preparation of morpholinyl-*o*-nitroanilines.

**Scheme 3:**
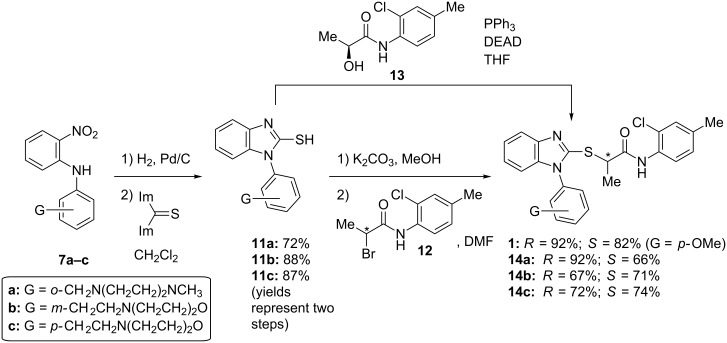
Asymmetric synthesis of (*R*)- and (*S*)-isomers by using two different approaches.

**Synthesis.** The compounds were prepared by modifications of our previously reported routes. Briefly, nitroanilines **7** were obtained by one of two routes: *ortho-*nitrobenzaldehyde (**2**) was treated with *N*-methylpiperazine (**3**) in the presence of sodium triacetoxyborohydride to give nitrobenzene **4**, which was reduced under hydrogenation conditions to provide aniline **5**. *o*-Fluoronitrobenzene (**6**) was coupled with the previously formed aniline under S_N_Ar conditions to furnish anilino nitrobenzene **7a** (Scheme 1). Alternatively, *meta-* and *para-*nitrophenylethanols **8** were combined with *o*-fluoronitrobenzene (**6**) to deliver *o*-nitroanilines **9**. The hydroxy groups of **9** were activated as the *p*-nitrobenzenesulfonates **10** and displaced with morpholine to give *o*-nitroanilines **7b** and **7c** (Scheme 2). Hydrogenation was used to reduce *o*-nitroanilines **7** followed by cyclization using thiocarbonyldiimidazole to yield benzothiazoles **11**. In the case of the racemates, these were combined with α-bromopropionamide **12** to afford racemic **14** (Scheme 3).

**Scheme 4:**
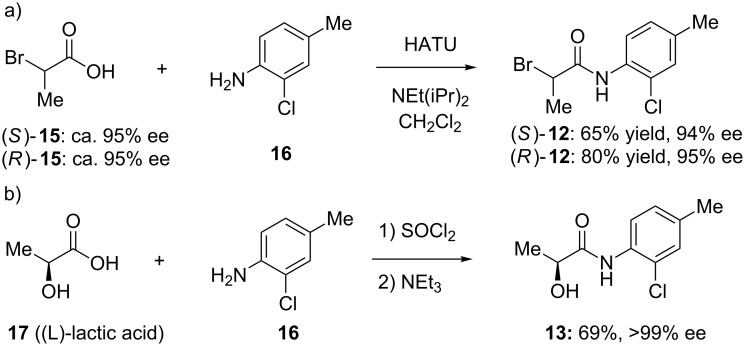
Preparation of chiral propionamides by HATU-mediated coupling (a) or thionyl chloride-mediated coupling (b).

To address the issue of stereochemical control, we developed two strategies for preparing the compounds enantioselectively. The first approach utilized commercially available (*R*)- or (*S*)-2-bromopropionic acid (**15**) from the chiral pool in a two-step sequence (Scheme 4a). The first step involved amide bond formation of 2-bromopropionic acid (**15**) with 2-chloro-4-methylaniline (**16**) by using HATU (*O*-(7-azabenzotriazol-1-yl)-*N,N,N',N'-*tetramethyluronium hexafluorophosphate), a reagent known to limit racemization in peptide coupling reactions [16]. The enantiomeric excesses of the starting materials were ca. 95%. No erosion of stereochemistry in the products **12** was observed as determined by chiral HPLC. Recrystallization of **12** from ethanol did not increase the enantiomeric excess of the α-bromoamide. Interestingly, the final product can be achieved in both high yield and ee by adding the preformed potassium salt of the 2-mercaptobenzimidazole **11** to (*S*)- or (*R*)-α-bromopropionamides **12**. Using a substoichiometric amount of potassium carbonate (0.9 equiv) in warm methanol, followed by removal of solvents under vacuum, provided a convenient route to the potassium salt of **11**. The salt was suspended in anhydrous DMF and added dropwise to a cold (0 °C) solution of the previously formed bromides, to give the desired products **1** and **14** in high yield (80–90%) with little to no loss in enantiomeric excess. Isolating the potassium salt proved necessary, as attempts at preforming the potassium salt in dry DMF, followed by addition to the chiral α-bromoamide, led to ee’s of ca. 60%. Although both chiral acids are readily available from commercial sources, their enantiomeric excesses seem to be limited to ca. 95%. For these studies, ee’s >90% were sufficient; if material with higher enantiopurity is needed, we note that one recrystallization of (*S*)-**1** from ethyl ether increases the enantiomeric excess from 94% to 97.4%.

In an alternative approach that provides material with high enantiomeric excess (>98% ee), we utilized the Mitsunobu reaction of 2-mercaptobenzimidazoles with an amide obtained from (L)-lactic acid (**17**). Using one equivalent each of 2-mercaptobenzimidazole and α-hydroxyamide **13** (prepared from thionyl chloride-mediated coupling of (L)-lactic acid (**17**) and 2-chloro-4-methylaniline (**16**) [17] (Scheme 4b)) in the presence of a slight excess of triphenylphosphine and diethyl azodicarboxylate, we acquired the desired products **14** in good yield and ee. This methodology is attractive because of the high ee that can be obtained from the inexpensive and readily available (L)-lactic acid (**17**). However, the enantiomeric (D)-lactic acid is substantially more expensive, and as seen below, the products arising from (L)-lactic acid are less active, making this route less attractive than the route utilizing chiral α-bromopropionamides **12**.

**Figure 2:**
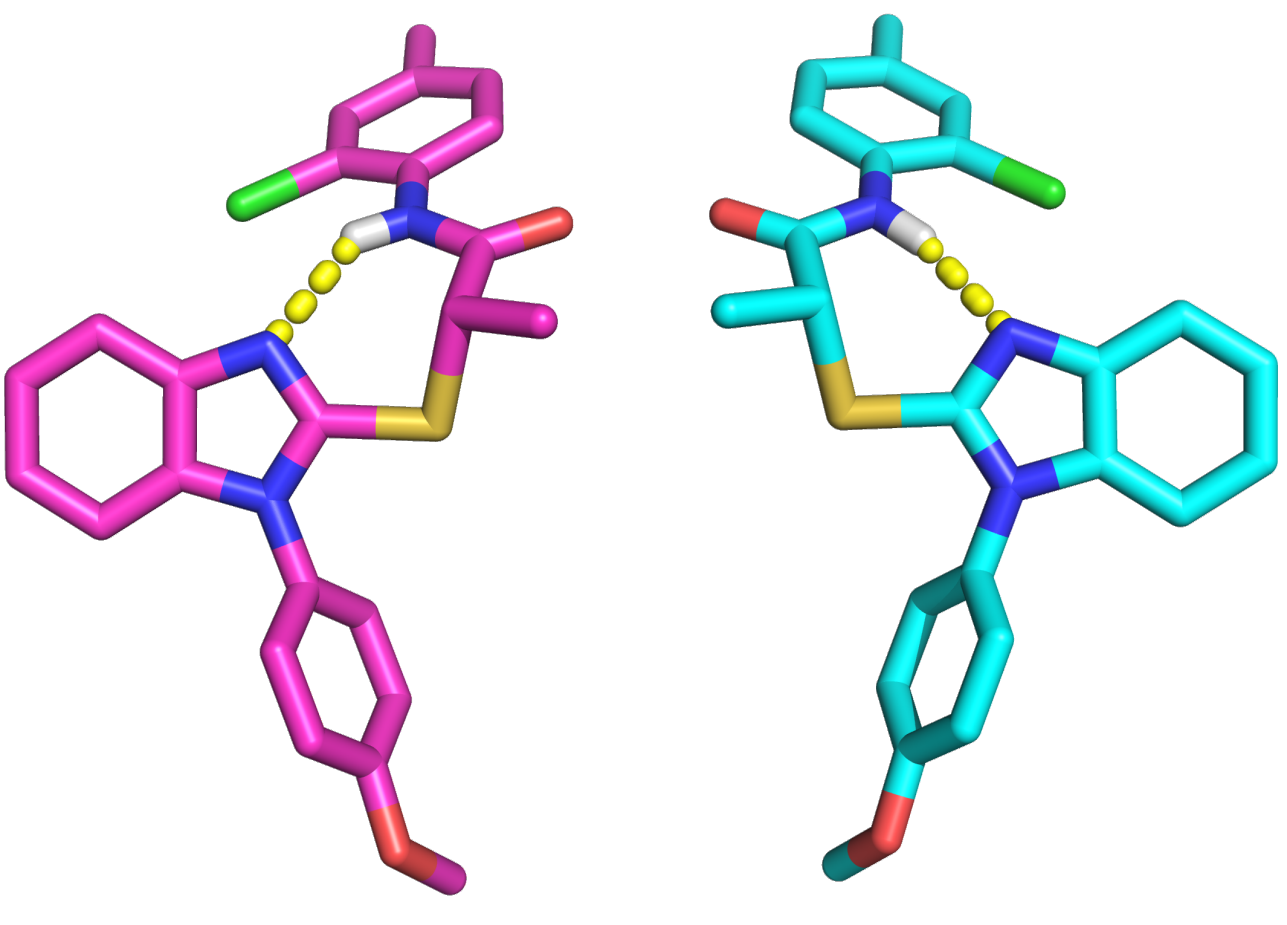
Renderings of crystal structures of (*S*)*-* (left, magenta) and (*R*)*-* (right, cyan) enantiomers of **1**.

Crystal structures of (*R*)- and (*S*)-**1** obtained by crystallization from ethyl ether allowed us to unambiguously assign the absolute configuration of each enantiomer. Shown in Figure 2 are the (*S*)-enantiomer (left, magenta) and (*R*)-enantiomer (right, cyan) of **1**. It is interesting to note that the hydrogen-bond formed between the amide N–H and the unsubstituted benzimidazole nitrogen in these crystal structures results in pseudo seven-membered rings. Whether this conformation is biologically relevant is unknown.

### Structure–activity relationships

**Figure 3:**
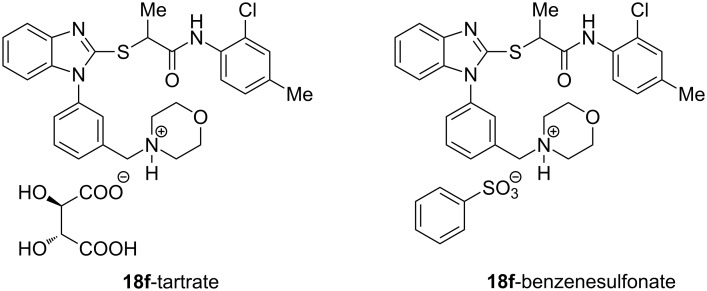
L-Tartaric acid salt (**18f**-tartrate) and benzenesulfonic acid salt (**18f**-benzenesulfonate) of **18f**.

A small set of compounds was synthesized based on variations of **1** by replacing the *p*-methoxyphenyl group with other substituted phenyl rings or heterocyclic rings. The compounds were initially assayed in two screening assays: (1) a measles virus cytopathic effect (CPE) reduction assay [12] and (2) a solubility assay based on laser nephelometry [18]. Unfortunately, compounds with the highest aqueous solubilities (>100 µg/mL) had the poorest antiviral activity (i.e., **18a** and **18e**, Table 1). To explore the possibility of increasing solubility by salt formation, the L-tartaric acid salt and benzenesulfonic acid salts of the most active compound **18f** were synthesized (Figure 3) and subjected to solubility testing. However, these salts failed to improve solubility compared with the parent. Several compounds exhibited moderate antiviral activities but poor to moderate aqueous solubilities (i.e., **1**, **18b**, **18d**, and **18f**, Table 1). Since **14a**, **14b**, and **14c** showed good antiviral activities, as well as moderate aqueous solubilities, we decided to examine the broader antiviral activities of these compounds and to determine what, if any, effect the stereocenter present in each of these compounds may cause.

**Table 1:** In vitro screening of analogues of **1**.

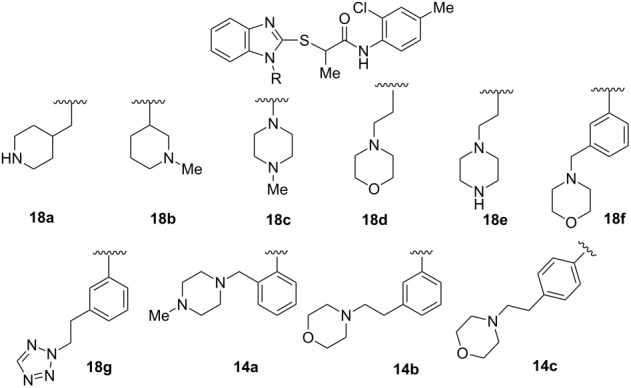					

Compd.	MeV^a^ (CPE, µM)	Aqueous solubility^b^ (µg/mL at pH 7.4)	Compd.	MeV^a^ (CPE, µM)	Aqueous solubility^b^ (µg/mL at pH 7.4)

**1**	3.1	<15	**18f-**tartrate	ND^c^	<15
**18a**	>75	140	**18f-**benzene sulfonate	ND	<15
**18b**	>0.29	19	**18g**	0.05^d^	<15
**18c**	>75	<15	**14a**	0.179	20
**18d**	4.9	22	**14b**	0.20	15
**18e**	>75	120	**14c**	0.6	25
**18f**	0.27	<15			

^a^50% inhibitory concentrations were calculated by using the variable-slope (four parameters) nonlinear regression-fitting algorithm embedded in the Prism 5 software package (GraphPad Software). Values represent averages of four experiments; highest concentration assessed, 75 μM. ^b^Determined through laser nephelometry; ^c^ND. Not determined. ^d^Virus yield reduction assay was used.

We were motivated by the results of these two assays to more completely characterize the antiviral activities and solubility parameters of the most promising compounds. We assayed the compounds in three additional biological assays: (1) a firefly luciferase minireplicon assay whose output is driven by infection with influenza A/sw/Texas/2009 (WSN); (2) an assay using a renilla luciferase reporter embedded as an additional transcription unit in the genome of a measles virus (MeV) recombinant; and (3) a colorimetric assay that measures reduction of MTT (3-(4,5-dimethylthiazol-2-yl)-2,5-diphenyltetrazolium bromide) as a surrogate for general cytotoxicity, reported here as CC_50_. Additionally, the aqueous solubilities of the compounds were measured by using laser nephelometry at pH 3.0, 5.0, and 7.4 (Table 2).

**Table 2:** Antiviral potencies and solubilities for **1** and analogues.

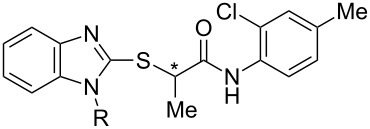							

Compd.	R	EC_50_ (nM)^a^		Solubility (µg/mL)^b^			CC_50_ (nM)^c^
		MeV	WSN	pH 3.0	pH 5.0	pH 7.4	

**1** (*S*)-**1** (*R*)-**1**	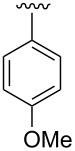	31 37 880	13 5 160	<15	<15	<15	>50000 >50000 >50000
**14b** (*S*)-**14b** (*R*)-**14b**	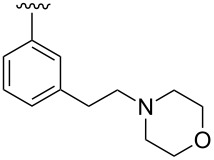	13 11 210	0.3 1 110	158	33	15	>50000 >50000 >50000
**14c** (*S*)-**14c** (*R*)-**14c**	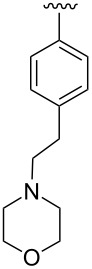	110 180 530	43 36 120	212	51	25	ND^d^ ND ND
**14a** (*S*)-**14a**^e^ (*R*)-**14a**^e^	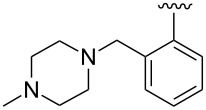	41 68 470	8 4 120	>300	92	20	ND 25000 ND

^a^50% effective concentrations were calculated by using the variable-slope (four parameters) nonlinear regression-fitting algorithm embedded in the Prism 5 software package (GraphPad Software). Values represent averages of four experiments; highest concentration assessed 75 μM. ^b^Determined through laser nephelometry. ^c^Determined through MTT assay, highest concentration assessed 50 μM. ^d^ND: Not determined. ^e^Assayed as a 1:1 mixture of atropisomers. Details of the separation of the atropisomers will be discussed elsewhere.

Analysis of the data reveals several trends. First, with the exception of a single case in which enantiomer potencies are similar (**14c**, ΔEC_50_ < 3-fold), six other comparisons reveal the (*S*)-enantiomer to be more active than the (*R*)-form by 7 to 110-fold. Although we do not know the identity of the specific biological target(s), the enantio-discrimination implies to us that the molecules bind in a well-defined binding pocket that is able to accommodate the *S*-enantiomer more readily than the (*R*)-enantiomer.

We note that the activity trends for the (*S*)- and (*R*)-enantiomers against the measles and WSN influenza strain are qualitatively similar. However, for antiviral potency differences between the racemate and the (*S*)-enantiomer, the attenuation is indistinguishable under the testing conditions. Accordingly, we attribute the assay discrepancy to the inherent variability in the assay system.

While the compounds appear to be active against both influenza and measles virus, they are somewhat more active against the influenza virus strain (WSN) than against the measles virus. Among the compounds surveyed, (*S*)-**14b** is the most potent compound, with EC_50_ values of 1–11 nM against the two viruses. Because the CC_50_ values of the compounds are greater than 50 µM, the upper limit of the assay, we assume that the compounds are not generally cytotoxic, giving selectivity indices (CC_50_/EC_50_) for the active enantiomers of at least 10^3^–10^4^.

We have also assayed the most active compounds in a human parainfluenza viral (HPIV) titer assay based on plaque assay titration. The values for (*S*)-**1**, (*S*)-**14a**, (*S*)-**14b**, and (*S*)-**14c** are 80, 13, 80, and 11 nM, respectively. These data further corroborate the broad-spectrum activity of these compounds.

Lastly, the solubilities of the compounds have been improved relative to that of **1**. At all pH values examined, the aqueous solubility of **1** was below the limits of detection of the nephelometry assay (i.e., <15 µg/mL). However, compounds bearing basic amine functionalities have improved solubilities relative to **1**, particularly at acidic pH values, but also at pH 7.4.

## Conclusion

We have extended our previously published work on host-directed inhibitors of myxovirus replication by preparing analogues that positively address the poor aqueous solubility of **1** (JMN3-003) while simultaneously improving its potency. The compounds presented here furnish EC_50_ values as low as 1 nM with aqueous solubilities ranging from 15–25 µg/mL at pH 7.4 to >300 µg/mL at pH 3.0. Additionally, we have developed two complementary methods for the synthesis of each of the enantiomers of the compounds discussed and have unequivocally demonstrated that the (*S*)-enantiomer is more active in this series than the (*R*)-enantiomer. Further work from our laboratories regarding the in vivo efficacy of these compounds is underway.

## Supporting Information

Contains detailed synthetic procedures and characterization data for molecules described herein, a more detailed description of the laser nephelometry assay, and data tables for the crystal structures of (*S*)-**1** and (*R*)-**1**.

File 1 Detailed synthetic procedures and characterization data.
