# Supplementary material for: Asymmetric synthesis of host-directed inhibitors of myxoviruses
Source: Beilstein J Org Chem. 2013 Jan 30;9:197–203. doi: 10.3762/bjoc.9.23 (PMC3566758; doi:10.3762/bjoc.9.23)
Supplement: File 1 — Detailed synthetic procedures and characterization data. [file Beilstein_J_Org_Chem-09-197-s001.pdf]

Supporting Information

for

Asymmetric synthesis of host-directed inhibitors of myxoviruses

Terry W. Moore[1], Kasinath Sana[1], Dan Yan[2,3], Pahk Thepchatri[1], John M. Ndungu[1], Manohar T. Saindane[1], Mark A. Lockwood[1], Michael G. Natchus[1], Dennis C. Liotta[1,4], Richard K. Plemper[2,3,5], James P. Snyder[1,4] and Aiming Sun[*1,§]

Address:[1]Emory Institute for Drug Development, Emory University, 1515 Dickey Drive, Atlanta, GA 30322, USA, [2]Department of Pediatrics, Emory University School of Medicine, 2015 Uppergate Drive, Atlanta, GA 30322, USA, [3]Children's Healthcare of Atlanta, 2015 Uppergate Drive, Atlanta, GA 30322, USA, [4]Department of Chemistry, Emory University, 1515 Dickey Drive, Atlanta, GA 30322, USA and [5]Department of Microbiology & Immunology, Emory University School of Medicine, 1510 Clifton Road, Atlanta, GA, 30322, USA

[§]Phone: 404-712-8680; Fax: 404-727-6689

Email: Aiming Sun - asun2@emory.edu

*Corresponding author

Detailed synthetic procedures and characterization data

Table of contents:

General considerations	S2
Synthetic procedures	S2–S12
Tables for crystal structure of (*S*)-**1**	S13–S24
Tables for crystal structure of (*R*)-**1**	S25–S36
Procedure for laser nephelometry studies	S36
^{1}H NMR spectra of assayed compounds	S37–S52
Reference	S52

General Considerations. Unless otherwise noted, all materials were obtained from commercial suppliers and used without purification. Dry organic solvents (DriSolv) were purchased from EMD Chemicals and packaged under nitrogen in Sure Seal bottles. Reactions were monitored using thin-layer chromatography on 250 μm plates or using Agilent 1100 series LC/MS with UV detection at 254 nm and a low resonance electrospray mode (ESI). Purification of title compounds was accomplished by flash column chromatography using silica gel 60 (particle size 0.04–0.063 mm, 230–400 mesh) or liquid chromatography on a Biotage SP4 purification system with normal phase silica gel. ^{1}H NMR spectra were recorded on a Varian spectrometer (400 MHz) at ambient temperature. Chemical shifts are reported in ppm relative to $CDCl_3$ or CD_3OD and coupling constants (J) are reported in hertz (Hz). Solvents for NMR were deuteriochloroform ($CDCl_3$) (residual shifts: δ 7.26 for ^{1}H and δ 77.7 for ^{13}C) and deuteriomethanol (CD_3OD) (residual shift: δ 3.31 for ^{1}H). The residual shifts were taken as internal references and reported in parts per million (ppm). Purity of final compounds was ≥95% based on analytical HPLC and NMR analysis.

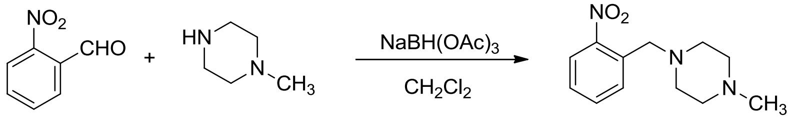

1-Methyl-4-(2-nitrobenzyl)piperazine (4). To a solution of 2-nitrobenzaldehyde (**3**, 12.0 g, 79.0 mmol) in CH_2Cl_2 (150 mL) was added 1-methylpiperazine (**2**, 9.54 g, 95.0 mmol) and one drop of acetic acid, followed by sodium triacetoxyborohydride (21.8 g, 103 mmol). The reaction mixture was stirred at rt for 18 h, quenched with sat. ammonium chloride solution, and stirred for 30 min. The organic layer was separated and washed once with water, dried over $MgSO_4$, and concentrated to give 17.7 g (94% yield) of a colorless oil, which was used in the next step without further purification. ^{1}H NMR (400 MHz, $CDCl_3$) δ 7.73 (dd, J = 7.6, 0.8 Hz, 1H), 7.49-7.53 (m, 1H), 7.38-7.43 (m, 2H), 3.40 (s, 2H), 2.81 (br s, 4H), 2.65 (t, J = 5.0 Hz, 4H), 2.51 (s, 3H). m/z (ESI) = 236.2 $[M + H]^+$; TLC 100% EtOAc R_f = 0.7.

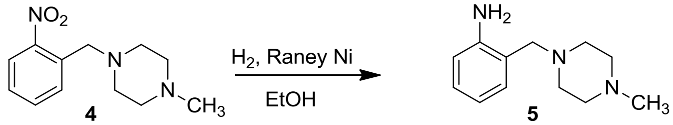

2-((4-methylpiperazin-1-yl)methyl)aniline (5). A 500 mL Parr reaction vessel was charged with 1-methyl-4-(2-nitrobenzyl)piperazine (**4**, 2.77 g, 11.8 mmol), ethanol (100 mL), and 0.25 g of Raney Nickel. The reaction vessel was flushed with hydrogen three times, pressurized with hydrogen to 40 psi, and allowed to react at ambient temperature. Additional hydrogen was added as the pressure dropped. After one hour, the reaction mixture was filtered and concentrated under reduced pressure. The residue was taken up in 100 mL of ethyl acetate and washed with dilute NH_4OH solution, water, and brine. The organic layer was dried over magnesium sulfate, filtered and concentrated under reduced pressure to yield 2-((4-methylpiperazin-1-yl)methyl)aniline (**5**, 2.10 g, 10.2 mmol, 87% yield) as a clear colorless oil, which solidified to an off-white solid after drying in vacuo. ^{1}H NMR (400 MHz, $CDCl_3$) δ 6.96-

7.09 (m, 2H), 6.60-6.66 (m, 2H), 4.65 (br s, 2H), 3.49 (s, 2H), 2.47 (br s, 8H), 2.26 (s, 3H). *m*/*z* (ESI) = 206.2 $[M + H]^+$

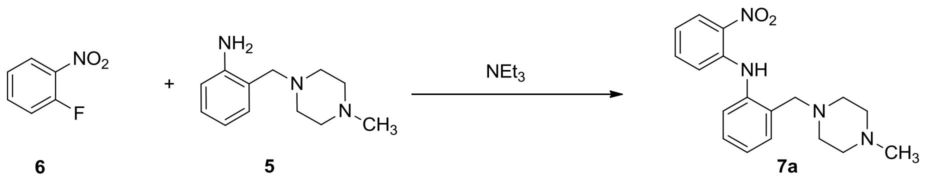

6 5 7a

2-((4-methylpiperazin-1-yl)methyl)-*N*-(2-nitrophenyl)aniline (7a). In a thick-walled 15 mL pressure vessel with a screw-top Teflon cap was added 2-((4-methylpiperazin-1-yl)methyl)aniline (**5**, 0.145 g, 0.709 mmol), triethylamine (0.148 mL, 1.06 mmol) and 1-fluoro-2-nitrobenzene (**6**, 0.075 mL, 0.709 mmol). The vessel was sealed and heated at 160 °C for 2 h. The reaction mixture was cooled to ambient temperature. The residue was taken up in CH_2Cl_2 and purified using silica gel flash chromatography (0–15% MeOH in CH_2Cl_2). The fractions were combined and concentrated to yield 2-((4-methylpiperazin-1-yl)methyl)-*N*-(2-nitrophenyl)aniline (**7a**, 0.100 g, 0.306 mmol, 43% yield) as an orange solid. ^{1}H NMR (400 MHz, $CDCl_3$) δ 10.43 (s, 1H), 8.18 (dd, *J* = 8.4, 1.6 Hz, 1H), 7.23-7.43 (m, 5H), 7.05 (td, *J* = 7.6, 1.2 Hz, 1H), 6.76 (ddd, *J* = 8.4, 6.8, 1.2 Hz, 1H), 3.48 (s, 2H), 2.56 (br s, 8H), 2.33 (s, 3H). *m*/*z* (ESI) = 327.2 $[M + H]^+$

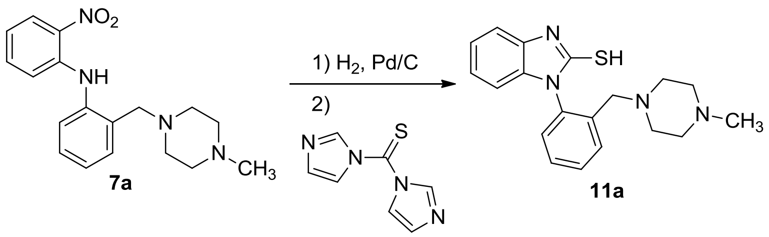

7a 11a

1-(2-((4-methylpiperazin-1-yl)methyl)phenyl)-1*H*-benzo[*d*]imidazole-2(3*H*)-thione (11a). A 250 mL Parr reaction vessel was charged with 10% palladium on carbon (0.5 g), ethanol (50 mL), and 2-((4-methylpiperazin-1-yl)methyl)-*N*-(2-nitrophenyl)aniline (**7a**, 16.0 g, 49.0 mmol) dissolved in additional EtOH (200 mL). The flask was flushed with hydrogen three times and pressurized to 50 psi. The reaction mixture was shaken at ambient temperature, and additional hydrogen was added as consumed until the reaction was complete. The reaction mixture was filtered and concentrated under reduced pressure to yield a dark oil. LC–MS was used to confirm presence of product (*m*/*z* 297.2 $[M + H]^+$), and, because of the instability of the product, it was used immediately in the following step without further characterization.

A 500 mL round-bottomed flask was charged with the previously prepared N^1-(2-((4-methylpiperazin-1-yl)methyl)phenyl)benzene-1,2-diamine (13.4 g, 45.2 mmol) and CH_2Cl_2 (200 mL); the mixture was stirred until dissolved. 1,1'-thiocarbonyldiimidazole (9.67 g, 54.2 mmol) was added in one portion and stirred at ambient temperature for one hour. The reaction mixture was quenched with water. The layers were separated, and organic layer was washed with saturated $NaHCO_3$ and brine, dried over sodium sulfate, and concentrated under reduced pressure to yield a dark oil. The oil was purified by silica gel column chromatography (0–15% MeOH/CH_2Cl_2). The fractions containing product were combined and concentrated

under reduced pressure to yield 1-(2-((4-methylpiperazin-1-yl)methyl)phenyl)-1H-benzo[*d*]imidazole-2(3H)-thione (11.0 g, 32.5 mmol, 72% yield) as a tan solid. ^{1}H NMR (400 MHz, $CDCl_3$) δ 7.68 (s, 1H), 7.60 (dd, *J* = 7.4, 1.5 Hz, 1H), 7.53-7.39 (m, 2H), 7.32 (dd, *J* = 7.5, 1.5 Hz, 1H), 7.22-7.10 (m, 2H), 7.10-7.02 (m, 2H), 6.67 (d, *J* = 7.9 Hz, 1H), 3.57 (d, *J* = 14.0 Hz, 1H), 3.29 (d, *J* = 14.0 Hz, 1H), 2.46-1.90 (br s, 8H) 2.19 (s, 3H). ^{13}C NMR (101 MHz, $CDCl_3$) δ 168.7, 137.4, 134.9, 134.3, 131.1, 130.9, 129.6, 129.0, 128.4, 123.2, 122.5, 110.0, 109.7, 59.2, 54.5, 52.7, 45.4. *m*/*z* (ESI) = 339.2 $[M + H]^+$

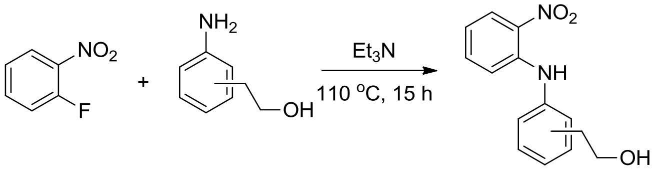

6 **8a** = *m*- **8b** = *p*- **9a** = *m*-, 77% **9b** = *p*-, 62%

2-(3-((2-Nitrophenyl)amino)phenyl)ethanol (9a): 2-(3-aminophenyl)ethanol (**8a**, 3.28 g, 23.9 mmol), 1-fluoro-2-nitrobenzene (**6**, 3.0 mL, 28.7 mmol) and Et_3N (4.0 mL, 28.7 mmol) were mixed in a sealed tube and heated at 110 °C for 15 h. Then the mixture was dissolved in CH_2Cl_2 and washed with water. The combined organic was washed with brine, dried over Na_2SO_4 and concentrated. Chromatographic purification using 25–30% ethyl acetate in hexanes provided **9a** (4.75 g, 77%) as a reddish brown solid. ^{1}H NMR (300 MHz, $CDCl_3$) *δ* 9.48 (s, 1 H), 8.20 (d, *J* = 8.7 Hz, 1H), 7.39-7.33 (m, 2H), 7.45 (d, *J* = 8.7 Hz, 1H), 7.16 (d, *J* = 6.3 Hz, 1H), 7.15 (s, 1H), 7.12 (d, *J* = 7.5 Hz, 1H), 6.77 (t, *J* = 7.5 Hz, 1H), 3.90 (t, *J* = 6.3 Hz, 2H), 2.89 (t, *J* = 6.3 Hz, 2H). *m*/*z* (ESI) $[M + H]^+$: 259.0.

2-(4-((2-Nitrophenyl)amino)phenyl)ethanol (9b): Following the same procedure as for the preparation of **9a**, using 2-(4-aminophenyl)ethanol (**8b**, 2.05 g, 15.0 mmol) as substrate, **9b** (2.4 g, 62%) was obtained as a reddish brown solid. ^{1}H NMR (300 MHz, $CDCl_3$) *δ* 9.47 (s, 1 H), 8.20 (d, *J* = 8.7 Hz, 1H), 7.38-7.18 (m, 6H), 6.76 (t, *J* = 7.5 Hz, 1H), 3.90 (t, *J* = 6.3 Hz, 2H), 2.90 (t, *J* = 6.3 Hz, 2H). *m*/*z* (ESI) $[M + H]^+$: 259.0.

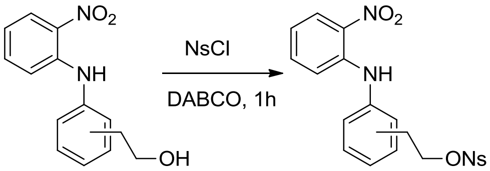

9a = *m*-, 77% **9b** = *p*-, 62% **10a** = *m*-, 87% **10b** = *p*-, 93%

3-((2-Nitrophenyl)amino)phenethyl 4-nitrobenzenesulfonate (10a): To a stirred solution of **9a** (4.2 g, 16.3 mmol) and 1,4-diazabicyclo[2.2.2]octane (2.74 g, 24.4 mmol) in CH_2Cl_2 (65 mL) at 0 °C was added 4-nitrobenzenesulfonyl chloride (5.04 g, 22.8 mmol) in portions over a 5 min period. The reaction mixture was then stirred at room temperature for 1 h. Water (50 mL) was

added and the resulting mixture was stirred for 10 min. The organic layer was separated, dried over Na_2SO_4, filtered and concentrated under reduced pressure. Chromatographic purification using 20-25% ethyl acetate in hexanes provided **10a** (6.3 g, 87%) as a reddish brown solid. ^{1}H NMR (300 MHz, $CDCl_3$) δ 9.40 (s, 1 H), 8.32 (d, J = 8.7 Hz, 2H), 8.16 (d, J = 8.7 Hz, 1H), 7.99 (d, J = 8.7 Hz, 2H), 7.39 (t, J = 7.8 Hz, 1H), 7.33-7.15 (m, 3H), 7.03 (s, 1H), 6.97 (d, J = 7.2 Hz, 1H), 6.80 (t, J = 7.5 Hz, 1H), 4.36 (t, J = 6.3 Hz, 2H), 3.02 (t, J = 6.3 Hz, 2H). *m/z* (ESI) $[M + H]^+$: 444.1.

4-((2-nitrophenyl)amino)phenethyl 4-nitrobenzenesulfonate (10b): Following the same procedure as for the preparation of **10a**, using **9b** (2.24 g, 8.7 mmol) as substrate, **10b** (3.57 g, 93%) was obtained as a reddish brown solid. ^{1}H NMR (300 MHz, $CDCl_3$) δ 9.41 (s, 1 H), 8.30 (d, J = 8.7 Hz, 2H), 8.17 (d, J = 8.7 Hz, 1H), 7.99 (d, J = 8.7 Hz, 2H), 7.39-7.09 (m, 6H), 6.80 (t, J = 7.5 Hz, 1H), 4.36 (t, J = 6.3 Hz, 2H), 3.02 (t, J = 6.3 Hz, 2H). *m/z* (ESI) $[M + H]^+$: 444.1.

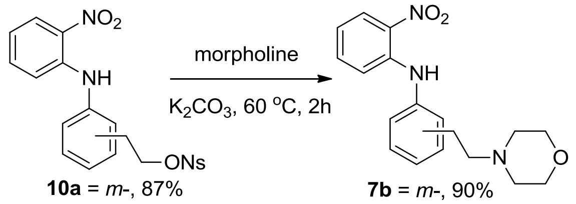

10a = *m*-, 87%
10b = *p*-, 93%
7b = *m*-, 90%
7c = *p*-, 95%

***N*-(3-(2-Morpholinoethyl)phenyl)-2-nitroaniline (7b):** To a stirred mixture of **10a** (3.5 g, 7.9 mmol) and K_2CO_3 (1.3 g, 9.5 mmol) in DMF (18 mL) at room temperature was added morpholine (1.5 mL, 15.8 mmol). The reaction mixture was then heated to 60 °C and stirred at that temperature for 2 h. Then the reaction mixture was concentrated, dissolved in ethyl acetate, and washed with water. The aqueous phase was extracted with ethyl acetate and the combined organic phase was washed with water and brine, dried over Na_2SO_4 and concentrated. Chromatographic purification using 3–5% MeOH in CH_2Cl_2 afforded **7b** (2.32 g, 90%) as a reddish brown solid. ^{1}H NMR (300 MHz, $CDCl_3$) δ 9.47 (s, 1H), 8.18 (d, J = 8.4 Hz, 1H), 7.38-7.23 (m, 3H), 7.11 (s, 1H), 7.06 (d, J = 7.2 Hz, 2H), 6.75 (t, J = 7.5 Hz, 1H), 3.72 (t, J = 4.5 Hz, 4H), 2.80 (t, J = 6.3 Hz, 2H), 2.58 (t, J = 6.3 Hz, 2H), 2.51 (t, J = 4.5 Hz, 4H). *m/z* (ESI) $[M + H]^+$: 328.1.

***N*-(4-(2-Morpholinoethyl)phenyl)-2-nitroaniline (7c):** Following the same procedure as for the preparation of **7b**, using **10b** (3.5 g, 7.9 mmol) as substrate, **7c** (2.45 g, 95%) was obtained as a reddish brown solid. ^{1}H NMR (300 MHz, $CDCl_3$) δ 9.45 (s, 1H), 8.18 (d, J = 8.4 Hz, 1H), 7.38-7.13 (m, 6H), 6.75 (t, J = 7.5 Hz, 1H), 3.71 (t, J = 4.5 Hz, 4H), 2.81 (t, J = 6.3 Hz, 2H), 2.60 (t, J = 6.3 Hz, 2H), 2.50 (t, J = 4.5 Hz, 4H). *m/z* (ESI) $[M + H]^+$: 328.1.

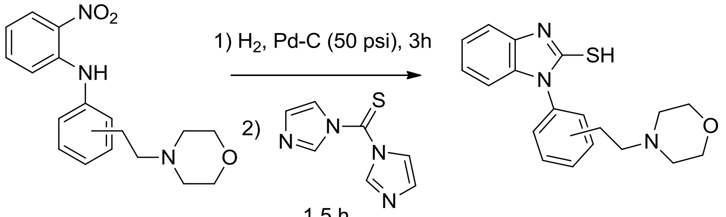

7b = *m*-, 90%
7c = *p*-, 95%
11b = *m*-, 88%
11c = *p*-, 87%

1-(3-(2-Morpholinoethyl)phenyl)-1*H*-benzimidazole-2-thiol (11b): 10% Pd/C (350 mg) was added to a solution of **7b** (2.3 g, 7.0 mmol) in MeOH: EtOAc (v/v 3:1, 48 mL) and the mixture was exposed to hydrogen at 40 psi for 3 h. The mixture was filtered through a pad of celite and the filtrate was concentrated to give *N*-(3-(2-morpholinoethyl)phenyl)benzene-1,2-diamine, which was taken to the next step without purification.
To a stirred solution of the previously formed *N*-(3-(2-morpholinoethyl)phenyl)benzene-1,2-diamine (7.0 mmol) in CH_2Cl_2 (50.0 mL) at 0 °C was added 1,1-thiocarbonyldiimidazole (1.87 g, 10.5 mmol), and the mixture was stirred for 1.5 h at room temperature. The organic layer was separated and washed with water (50.0 mL), dried over Na_2SO_4, filtered and concentrated. Chromatographic purification using 3–5% MeOH in CH_2Cl_2 afforded **11b** (2.1 g, 88% over two steps) as a colorless solid. ^{1}H NMR (300 MHz, $CDCl_3$) δ 7.52 (t, *J* = 8.4 Hz, 1H), 7.40-7.36 (m, 3H), 7.30-7.12 (m, 3H), 6.96 (d, *J* = 7.8 Hz, 1H), 3.76 (t, *J* = 4.5 Hz, 4H), 2.93 (t, *J* = 6.9 Hz, 2H), 2.70 (t, *J* = 6.9 Hz, 2H), 2.57 (br s, 4H). *m*/*z* (ESI) $[M + H]^+$: 340.1.

1-(4-(2-Morpholinoethyl)phenyl)-1*H*-benzimidazole-2-thiol (11c): Following the same procedure as for the preparation of **11b**, using **7c** (2.4 g, 7.3 mmol) as substrate, **11c** (2.2 g, 87% over two steps) was obtained as a colorless solid. ^{1}H NMR (300 MHz, $CDCl_3$) δ 7.45 (s, 4H), 7.30-7.11 (m, 3H), 6.95 (d, *J* = 7.8 Hz, 1H), 3.79 (t, *J* = 4.5 Hz, 4H), 2.94 (t, *J* = 6.9 Hz, 2H), 2.71 (t, *J* = 6.9 Hz, 2H), 2.60 (br s, 4H). *m*/*z* (ESI) $[M + H]^+$: 340.1.

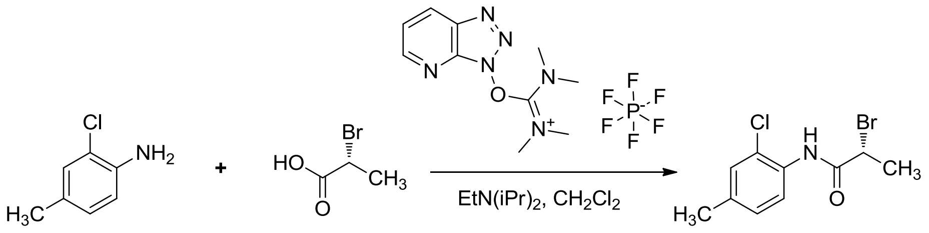

16 **15** (*R*)-**12**

(*R*)-2-bromo-*N*-(2-chloro-4-methylphenyl)propanamide ((*R*)-12). In dry CH_2Cl_2 (550 mL), (*R*)-2-bromopropanoic acid (*R*)-**15** (25.0 g, 163 mmol), 2-chloro-4-methylaniline (**16**, 21.0 g, 148 mmol) and 2-(3*H*-[1,2,3]triazolo[4,5-*b*]pyridin-3-yl)-1,1,3,3-tetramethyluronium hexafluorophosphate (56.5 g, 149 mmol) were dissolved. Diisopropylethylamine (19.2 g, 149 mmol) was added to this solution over a period of 30 min. The temperature of the reaction was maintained between 23 and 24 °C during the addition by occasional cooling with an ice-water bath. After the addition was complete, the mixture was stirred for 4 h at rt, after which time LC–MS showed the reaction to be >95% complete. The reaction mixture was filtered through a plug of celite, which was washed with 3 × 100 mL CH_2Cl_2. The residue was adsorbed onto 200 g of silica, and run through a short plug of silica using 25–50% EtOAc in hexanes (ca. 2 L). After removal of the solvent under reduced pressure from the fractions containing product, the solids were slurried in cold hexanes, collected by filtration, washed with cold hexanes, and dried to give 33.0 g (80% yield) of a white solid. ^{1}H NMR (400 MHz, $CDCl_3$) δ 8.58 (br s, 1H), 8.15 (d, *J* = 8.4 Hz, 1H), 7.19 (d, *J* = 1.6 Hz, 1H), 7.06 (d, *J* = 8.4, 1.6 Hz, 1H), 4.57 (q, *J* = 7.2 Hz, 1H), 2.29 (s, 3H), 1.96 (d, *J* = 7.2 Hz, 3H). ^{13}C NMR (100 MHz, $CDCl_3$) δ 167.4, 135.7, 131.6, 129.7, 128.6, 123.5, 121.4, 45.7, 23.4, 20.9. Chiral HPLC (Chiralcel OD-RH; 20% MeCN to 100% MeCN over 30 min; 1 mL/min; monitoring at 254 nm). UV(254 nm) purity: 97.7%. Ratio

of enantiomers: 97.4:2.6 (94.8% ee; t_r = 16.4, 17.1 min, respectively). HRMS-ESI (*m*/*z*): [M + H]$^+$ calcd. for $C_{10}H_{12}ONBrCl$, 275.97853; found, 275.97860.

(*S*)-2-bromo-*N*-(2-chloro-4-methylphenyl)propanamide ((*S*)-12). Following the same procedure as for the preparation of (*R*)-**12**, using (*S*)-2-bromopropanoic acid ((*S*)-**15**, 3.37 g, 22.0 mmol) and 2-chloro-4-methylaniline (**16**, 2.83 g, 20.0 mmol) as reactants, (*S*)-**12** (3.60 g, 65% yield) was obtained as white flakes. ^{1}H NMR (400 MHz, $CDCl_3$) δ 8.58 (br s, 1H), 8.15 (d, *J* = 8.4 Hz, 1H), 7.19 (d, *J* = 1.6 Hz, 1H), 7.06 (d, *J* = 8.4, 1.6 Hz, 1H), 4.57 (q, *J* = 7.2 Hz, 1H), 2.29 (s, 3H), 1.96 (d, *J* = 7.2 Hz, 3H). Chiral HPLC (Chiralcel OD-RH; 20% MeCN to 100% MeCN over 30 min; 1 mL/min; monitoring at 254 nm). UV(254 nm) purity: 97.7%. Ratio of enantiomers: 96.7:3.3 (93.4% ee; t_r = 16.5, 17.2 min, respectively). *m*/*z* (ESI) [M + H]$^+$: 278.0.

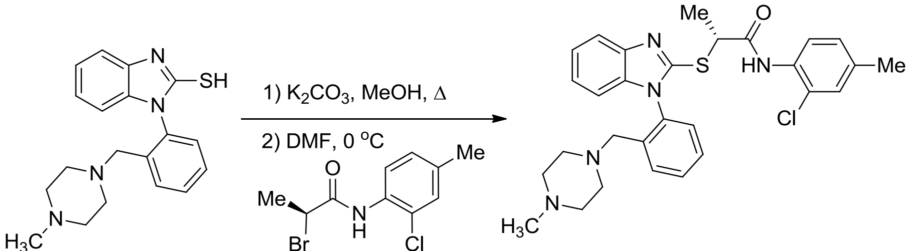

(*R*)-*N*-(2-chloro-4-methylphenyl)-2-((1-(2-((4-methylpiperazin-1-yl)methyl)phenyl)-1H-benzo[*d*]imidazol-2-yl)thio)propanamide ((*R*)-14a). In a 50 mL flask, 1-(2-((4-methylpiperazin-1-yl)methyl)phenyl)-1H-benzo[*d*]imidazole-2-thiol (**11a**, 2.00 g, 5.91 mmol) and potassium carbonate (0.778 g, 5.63 mmol) were suspended in 50 mL dry methanol with sonication and stirred at 45 °C for 2 h. The methanol was removed under reduced pressure and co-evaporated with dry acetonitrile. The resulting residue was dried under high vacuum overnight, suspended in 10 mL dry DMF, and added dropwise to a solution of (*S*)-2-bromo-*N*-(2-chloro-4-methylphenyl)propanamide ((*S*)-**12**, 1.71 g, 6.19 mmol) in 10 mL anhydrous DMF at 0 °C over 0.5 hours. The reaction mixture was stirred at 0 °C for 0.5 hours, and reaction progress was monitored by HPLC and TLC (10% MeOH in CH_2Cl_2; R_f of product = 0.32). The reaction mixture was poured into brine (100 mL) and CH_2Cl_2 (100 mL). The layers were separated, and the organic layer was washed twice more with 100 mL brine, dried over $MgSO_4$, filtered, concentrated, and dried under high vacuum overnight. After silica gel column chromatography (0 to 20% methanol in CH_2Cl_2, 40 mL/min, 80 g silica gel column, 22 mL fractions), the fractions containing product (fractions 13–27) were combined and concentrated under reduced pressure to give 2.76 g (92% yield) of a white foam. ^{1}H NMR (1:1 mixture of atropisomers, data given for one) ($CDCl_3$, 400 MHz) δ 10.46 (s, 1H), 8.20 (d, *J* = 5.2 Hz, 1H), 7.70 (d, *J* = 8.0 Hz, 1H), 7.60 (d, *J* = 7.2 Hz, 1H), 7.50 (td, *J* = 7.6, 1.2 Hz, 1H), 7.40 (td, *J* = 7.6, 1.6 Hz, 1H), 7.28-7.21 (m, 2H), 7.15–7.10 (m, 2H), 7.01 (dd, *J* = 8.4, 1.6 Hz, 1H), 6.86 (d, *J* = 8.0 Hz, 1H), 4.90 (q, *J* = 7.2 Hz, 1H), 3.21 (app q, *J* = 7.2 Hz, 2H), 2.3–2.05 (m, 8H), 2.25 (s, 3H), 2.17 (s, 3H), 1.59 (d, *J* = 7.6 Hz, 3H). Attempts to separate the enantiomers of **14a** using chiral HPLC (Chiralcel OD-RH) were unsuccessful. *m*/*z* (ESI) [M + H]$^+$: 534.2.

(*S*)-*N*-(2-chloro-4-methylphenyl)-2-((1-(2-((4-methylpiperazin-1-yl)methyl)phenyl)-1H-benzo[*d*]imidazol-2-yl)thio)propanamide ((*S*)-14a). Following the same procedure as for the

preparation of (*R*)-**14a**, using (*R*)-**12** (107 mg, 0.387 mmol) and **11a** (125 mg, 0.369 mmol) as reactants, (*S*)-**14a** (124 mg, 66% yield) was obtained as a white foam. ^{1}H NMR (1:1 mixture of atropisomers, data given for one) ($CDCl_3$, 400 MHz) δ 10.46 (s, 1H), 8.20 (d, *J* = 5.2 Hz, 1H), 7.70 (d, *J* = 8.0 Hz, 1H), 7.60 (d, *J* = 7.2 Hz, 1H), 7.50 (td, *J* = 7.6, 1.2 Hz, 1H), 7.40 (td, *J* = 7.6, 1.6 Hz, 1H), 7.28-7.21 (m, 2H), 7.15–7.10 (m, 2H), 7.01 (dd, *J* = 8.4, 1.6 Hz, 1H), 6.86 (d, *J* = 8.0 Hz, 1H), 4.90 (q, *J* = 7.2 Hz, 1H), 3.21 (app q, *J* = 7.2 Hz, 2H), 2.3–2.05 (m, 8H), 2.25 (s, 3H), 2.17 (s, 3H), 1.59 (d, *J* = 7.6 Hz, 3H). *m*/*z* (ESI) $[M + H]^+$: 534.2. Attempts to separate the enantiomers of **14a** using chiral HPLC (Chiralcel OD-RH) were unsuccessful.

(*S*)-*N*-(2-chloro-4-methylphenyl)-2-((1-(4-methoxyphenyl)-1*H*-benzo[*d*]imidazol-2-yl)thio)propanamide ((*S*)-1). Following the same procedure as for the preparation of (*R*)-**14a**, using (*R*)-**12** (357 mg, 1.29 mmol) and 1-(4-methoxyphenyl)-1*H*-benzo[*d*]imidazole-2-thiol (315 mg, 1.23 mmol), prepared as previously described [1], as reactants **(**(*S*)-**1**, 432 mg, 82% yield) was obtained as a white foam. ^{1}H NMR ($CDCl_3$, 400 MHz) δ 10.46 (s, 1H), 8.23 (d, *J* = 8.4 Hz, 1H), 7.71 (d, *J* = 8.0 Hz, 1H), 7.33-7.00 (m, 8H), 4.92 (q, *J* = 7.2 Hz, 1H), 3.87 (s, 3H), 2.25 (s, 3H), 1.61(d, *J* = 7.6 Hz, 3H). Chiral HPLC (Chiralcel OD-RH; 20% MeCN to 100% MeCN over 30 min; 1 mL/min; monitoring at 254 nm). UV(254 nm) purity: 99.5%. Ratio of enantiomers: 3.1:96.9 (93.8% ee; t_r = 26.7, 28.0 min, respectively). *m*/*z* (ESI) $[M + H]^+$: 452.0.

(*R*)-*N*-(2-chloro-4-methylphenyl)-2-((1-(4-methoxyphenyl)-1*H*-benzo[*d*]imidazol-2-yl)thio)propanamide (*R*)-1. Following the same procedure as for the preparation of (*R*)-**14a**, using (*S*)-**12** (357 mg, 1.29 mmol) and 1-(4-methoxyphenyl)-1*H*-benzo[*d*]imidazole-2-thiol (315 mg, 1.23 mmol), prepared as previously described [1] as reactants, (*R*)-**1** (487 mg, 92% yield) was obtained as a white foam. ^{1}H NMR ($CDCl_3$, 400 MHz) δ 10.46 (s, 1H), 8.23 (d, *J* = 8.4 Hz, 1H), 7.71 (d, *J* = 8.0 Hz, 1H), 7.33 (dd, *J* = 6.6, 2.2 Hz, 1H), 7.26 (app t, *J* = 8.0 Hz, 1H), 7.17 (app t, *J* = 8.0 Hz, 1H), 7.11-7.00 (m, 1.6 Hz, 5H), 4.92 (q, *J* = 7.2 Hz, 1H), 3.87 (s, 3H), 2.25 (s, 3H), 1.61(d, *J* = 7.2 Hz, 3H). Chiral HPLC (Chiralcel OD-RH; 20% MeCN to 100% MeCN over 30 min; 1 mL/min; monitoring at 254 nm). UV(254 nm) purity: 100%. Ratio of enantiomers: 96.9:3.1 (93.8% ee; t_r = 26.9, 28.3 min, respectively). *m*/*z* (ESI) $[M + H]^+$: 452.0.

(2*S*)-*N*-(2-chloro-4-methylphenyl)-2-((1-(3-(2-morpholinoethyl)phenyl)-1*H*-benzo[*d*]imidazol-2-yl)thio)propanamide ((*S*)-14b). Following the same procedure as for the preparation of (*R*)-**14a**, using (*R*)-**12** (320 mg, 1.16 mmol) and **11b** (375 mg, 1.11 mmol), (*S*)-**14b** (397 mg, 71% yield) was obtained as a white foam. ^{1}H NMR (400 MHz, $CDCl_3$) δ 10.42 (s, 1H), 8.24 (d, *J* = 8.8 Hz, 1H), 7.71 (d, *J* = 8.0 Hz, 1H), 7.46 (t, *J* = 7.8 Hz, 1H), 7.34 (d, *J* = 7.6 Hz, 1H), 7.29-7.12 (m, 6H), 7.01 (d, *J* = 8.0 Hz, 1H), 4.94 (q, *J* = 7.2 Hz, 1H), 3.72 (t, *J* = 4.4 Hz, 4H), 2.88 (t, *J* = 8.2 Hz, 2H), 2.66 (t, *J* = 7.8 Hz, 2H), 2.51 (br s, 4H), 2.24 (s, 3H), 1.62 (d, *J* = 7.6 Hz, 3H). Chiral HPLC (Chiralcel OD-RH; 35% MeCN to 45% MeCN in 0.1% TFA over 30 min; 1 mL/min; monitoring at 254 nm). Ratio of enantiomers: 2.1:97.9 (95.8% ee; t_r = 7.8, 8.6 min, respectively). *m*/*z* (ESI) $[M + H]^+$: 535.0.

(2*S*)-*N*-(2-chloro-4-methylphenyl)-2-((1-(4-(2-morpholinoethyl)phenyl)-1*H*-benzo[*d*]imidazol-2-yl)thio)propanamide ((*S*)-14c). Following the same procedure as for the preparation of (*R*)-**14a**, using (*R*)-**12** (85 mg, 0.309 mmol) and **11c** (100 mg, 0.295 mmol), (*S*)-**14c** (111 mg, 74% yield) was obtained as a white foam. ^{1}H NMR (400 MHz, $CDCl_3$) δ 10.41 (s, 1H), 8.22 (d, *J* = 8.0 Hz, 1H), 7.70 (d, *J* = 8.0 Hz, 1H), 7.41-7.12 (m, 8H), 7.01 (dd, *J* = 8.2, 1.8 Hz, 1H), 4.92 (q, *J* = 7.6 Hz, 1H), 3.77 (t, *J* = 4.6 Hz, 4H), 2.90 (t, *J* = 8.0 Hz, 2H), 2.66 (t, *J*

= 8.2 Hz, 2H), 2.55 (br s, 4H), 2.24 (s, 3H), 1.61 (d, J = 7.2 Hz, 3H). m/z (ESI) $[M+H]^+$: 535.2. Chiral HPLC (Chiralcel OD-RH; 10% MeCN to 70% MeCN over 30 min; 1 mL/min; monitoring at 254 nm). Ratio of enantiomers: 2.3:97.7 (95.4% ee; t_r = 20.5, 21.0 min, respectively).

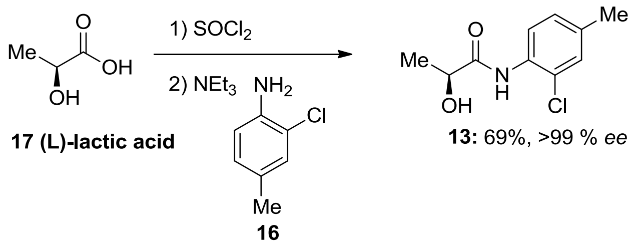

(2*S*)-*N*-(2-Chloro-4-methylphenyl)-2-hydroxypropanamide (13): To a stirred solution of (L)-(*S*)-lactic acid (**17**, 500 mg, 5.55 mmol) in dry THF (12 mL) at 0 °C, thionyl chloride (0.8 mL; 11.1 mmol) was added dropwise and stirring was continued for 3 h at 0 °C. Then triethylamine (1.55 mL, 11.1 mmol) was added into the reaction mixture and stirred at 0 °C for 20 minutes. 2-chloro-4-methylaniline (**16**, 0.72 mL, 5.83 mmol) was added and the mixture was stirred for 12 h at room temperature. THF was evaporated, the residue dissolved in 40 mL of CH_2Cl_2 and washed with 50 mL of 1% $NaHCO_3$ and then dried over Na_2SO_4, filtered and concentrated. Chromatographic purification using 20–25% ethyl acetate in hexanes afforded **13** (820 mg, 69%) as a white solid. ^1H NMR (300 MHz, $CDCl_3$): 9.11 (br s, 1H), 8.15 (d, J = 8.1 Hz, 1H), 7.14 (s, 1H), 7.11 (d, J = 8.1 Hz, 1H), 4.35 (q, J = 6.6 Hz, 1H), 4.07 (br s, 1H), 2.26 (s, 3H), 1.51 (d, J = 6.6 Hz, 3H). m/z (ESI) $[M + H]^+$: 214.0.

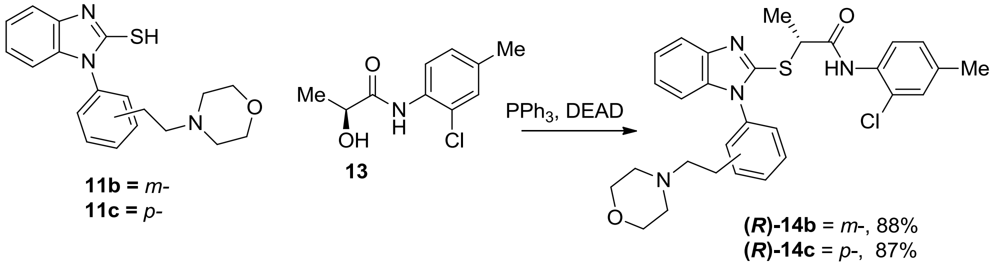

(2*R*)-*N*-(2-chloro-4-methylphenyl)-2-((1-(3-(2-morpholinoethyl)phenyl)-1*H*-benzo[*d*]imidazol-2-yl)thio)propanamide ((*R*)-14b): To a solution of **11b** (320 mg, 0.943 mmol), **13** (200 mg, 0.943 mmol) and triphenylphosphine (300 mg, 1.13 mmol) in THF (10.0 mL) at 0 °C, was added slowly diethyl azodicarboxylate (0.28 mL, 1.414 mmol). The reaction mixture was slowly allowed to warm ~20 °C over a period of 10 h, before water was added to quench the reaction. The mixture was extracted with CH_2Cl_2 and the organic layer was washed with brine, dried over Na_2SO_4, filtered and concentrated. Chromatographic purification using 20% iPrOH in hexanes was used to remove triphenylphosphine oxide and then the product was eluted with 3–5% MeOH in CH_2Cl_2 to afford (*R*)-**14b** (335 mg, 67%, >98% ee) as a white solid. ^1H NMR (300 MHz, $CDCl_3$) δ 10.43 (s, 1H), 8.25 (d, J = 8.7 Hz, 1H), 7.72 (d, J = 7.8 Hz, 1H), 7.48 (t, J = 8.1 Hz, 1H), 7.36 (d, J = 7.5 Hz, 1H), 7.29-7.12 (m, 6H), 7.02 (d, J = 8.4 Hz, 1H), 4.95 (q, J = 7.2 Hz, 1H), 3.74 (t, J = 4.5 Hz, 4H), 2.90 (t, J = 8.4 Hz, 2H), 2.66 (t, J = 8.4 Hz, 2H), 2.54 (br s, 4H), 2.26 (s, 3H), 1.63 (d, J = 7.2 Hz, 3H). m/z (ESI) $[M + H]^+$: 535.1; HPLC (Chiralcel OD-RH, linear gradients of 10–70% acetonitrile in 0.1% TFA water

buffer with a flow rate of 1 mL/min over 30 min and 254 nm UV detection) t_R 19.8 (*R*), 20.8 (*S*) min.

(2*R*)-*N*-(2-chloro-4-methylphenyl)-2-((1-(4-(2-morpholinoethyl)phenyl)-1*H*-benzo[*d*]imidazol-2-yl)thio)propanamide ((*R*)-14c): Following the same procedure as for the preparation of (*R*)-**14b**, using **11c** (300 mg, 0.884 mmol) as substrate, (*R*)-**14c** (340 mg, 72%, >97% ee) was obtained as a white solid. ^{1}H NMR (300 MHz, $CDCl_3$) δ 10.42 (s, 1H), 8.24 (d, *J* = 8.7 Hz, 1H), 7.72 (d, *J* = 7.8 Hz, 1H), 7.41-7.12 (m, 8H), 7.02 (d, *J* = 8.4 Hz, 1H), 4.93 (q, *J* = 7.2 Hz, 1H), 3.77 (t, *J* = 4.5 Hz, 4H), 2.91 (t, *J* = 8.4 Hz, 2H), 2.68 (t, *J* = 8.4 Hz, 2H), 2.56 (br s, 4H), 2.25 (s, 3H), 1.62 (d, *J* = 7.2 Hz, 3H). *m*/*z* (ESI) $[M+H]^+$: 535.1; HPLC (Chiralcel OD-RH, linear gradients of 10–70% acetonitrile in 0.1% TFA water buffer with a flow rate of 1 mL/min over 30 min and 254 nm UV detection) t_R 19.4 (*R*), 21.2 (*S*) min.

***N*-(2-chloro-4-methylphenyl)-2-((1(1-*t*-butoxycarbonyl-piperidin-4-ylmethyl)-1*H*-benzo[*d*]imidazol-2-yl)thio)propanamide (18a)**: Following the same procedure as for the preparation of **1**. The Boc group (415 mg, 0.76 mmol) was deprotected by TFA to obtain **18a** (296 mg, 87%) as a white solid. ^{1}H NMR (400 MHz, $CDCl_3$) δ 12.01 (br, 1H), 9.56 (s, 1H), 7.78 (s, 2H), 7.58 (m, 3H), 7.49 (d, *J* = 8.4 Hz, 1H), 7.07 (s, 1H), 7.01 (d, *J* = 8.4 Hz, 1H), 5.30-5.25 (m, 1H), 4.30 (d, *J* = 7.2 Hz, 3H), 3.45 (d, *J* = 12.0 Hz, 2H), 3.28 (d, *J* = 11.2 Hz, 2H), 2.97-2.94 (m, 2H), 2.76-2.73 (m, 2H), 2.38-2.33 (m, 2H), 2.30 (s, 3H). *m*/*z* (ESI) $[M + H]^+$: 443.2.

***N*-(2-chloro-4-methylphenyl)-2-((1-(1-methylpiperidin-3-yl)-1*H*-benzo[*d*]imidazol-2-yl)thio)propanamide (18b)**: Following the same procedure as for the preparation of **1**, **18b** was obtained as a white solid (48:52 mixture of diastereoisomers). ^{1}H NMR (400 MHz, $CDCl_3$) δ 10.42 (s, 1H), 10.36 (s, 1H), 8.20-8.17 (m, 1H), 7.63 (d, *J* = 8.0, 2H), 7.43 (d, *J* = 7.6, 3H), 7.24-7.14 (m, 2H), 7.06 (s, 1H), 7.00 (d, *J* = 8.0 Hz, 1H), 4.95-4.88 (m, 1H), 4.46-4.40 (m, 1H), 3.75-3.68 (m, 1H), 3.45(d, *J* = 13.2, 1H), 3.00-2.93 (m, 4H), 2.86 (s, 3H), 2.70 (t, *J* = 11.2, 1H), 2.33 (d, *J* = 6.0 Hz, 3H), 1.67 (d, *J* = 7.2, 3H). *m*/*z* (ESI) $[M + H]^+$: 446.2.

***N*-(2-chloro-4-methylphenyl)-2-((1-(4-methylpiperazinyl)-1*H*-benzo[*d*]imidazol-2-yl)thio)propanamide (18c)**: Following a similar procedure as for the preparation of **1**. **18c** was obtained as a white solid. ^{1}H NMR (400 MHz, $CDCl_3$) δ 10.22 (s, 1H), 8.22 (d, *J* = 8.0 Hz, 1H), 7.63 (d, *J* = 7.6 Hz, 1H), 7.51 (d, *J* = 8.0 Hz, 1H), 7.25-7.14 (m, 2H), 7.06 (d, *J* = 1.2 Hz, 1H), 6.99 (d, *J* = 10 Hz, 1H), 4.79 (app q, *J* = 7.2 Hz, 1H), 3.71 (m, 2H), 3.09 (app t, *J* = 14 Hz, 2H), 2.86 (app t, *J* = 10 Hz, 2H), 2.46-2.37 (m, 5H), 2.22 (s, 3H), 1.67 (d, *J* = 7.2 Hz, 3H). *m*/*z* (ESI) $[M + H]^+$: 444.2.

***N*-(2-chloro-4-methylphenyl)-2-((1-(2-morpholinoethyl)-1*H*-benzo[*d*]imidazol-2-yl)thio)propanamide (18d)**: Following a similar procedure as for the preparation of **1**. **18d** (50.3 mg, 94%) was obtained as a white solid. ^{1}H NMR (400 MHz, $CDCl_3$) δ 10.37 (s, 1H), 8.19 (d, *J* = 8.8 Hz, 1H), 7.66 (d, *J* = 8.0 Hz, 1H), 7.25-7.20 (m, 2H), 7.07 (s, 1H), 7.01 (d, *J* = 8.8 Hz, 1H), 4.89 (app q, *J* = 7.2 Hz, 1H), 4.14 (t, 6.8 Hz, 2H), 3.71 (app t, *J* = 4.4 Hz, 4H), 2.67 (t, *J* = 6.6 Hz, 2H), 2.46 (m, 4H), 2.23 (s, 3H), 1.68 (d, *J* = 7.6 Hz, 3H). *m*/*z* (ESI) $[M + H]^+$: 459.2.

***N*-(2-chloro-4-methylphenyl)-2-((1-(2-(piperazin-1-yl)ethyl)-1*H*-benzo[*d*]imidazol-2-yl)thio)propanamide (18e)**: Following a similar procedure as for the preparation of **1**. **18e** was

obtained as a white solid. ^{1}H NMR (400 MHz, $CDCl_3$) δ 10.42 (s, 1H), 8.24 (d, *J* = 8.0 Hz, 1H), 7.73 (d, *J* = 8.0 Hz, 1H), 7.52-7.46 (m, 2H), 7.34-7.26 (m, 4H), 7.21-7.12 (m, 2H), 7.03 (d, *J* = 8.8 Hz, 1H), 4.91 (app q, *J* = 7.2 Hz, 1H), 4.14 (t, 6.4 Hz, 2H), 3.05 (br. s, 4H), 2.77 (t, *J* = 6.0 Hz, 2H), 2.68 (m, 4H), 2.24 (s, 3H), 1.69 (d, *J* = 7.2 Hz, 3H). *m/z* (ESI) $[M + H]^+$: 458.2.

***N*-(2-chloro-4-methylphenyl)-2-((1-(3-((4-morpholinyl)methyl)phenyl)-1*H*-benzo[*d*]imidazol-2-yl)thio)propanamide (18f)**: Following a similar procedure as for the preparation of **1**, **18f** was obtained as a white solid. ^{1}H NMR (400 MHz, $CDCl_3$) δ 10.26 (s, 1H), 8.17 (d, *J* = 8.8 Hz, 1H), 7.66 (d, *J* = 6.0 Hz, 1H), 7.28-7.23 (m, 2H), 7.08 (d, *J* = 1.6 Hz, 1H), 7.02 (d, *J* = 8.8 Hz, 1H), 4.95 (app q, *J* = 7.2 Hz, 1H), 3.72 (t, *J* = 4.6 Hz, 4H), 3.58 (dd, *J* = 18, 14 Hz, 2H), 2.48 (br. s, 4H), 2.26 (s, 3H), 1.69 (d, *J* = 7.6 Hz, 3H). *m/z* (ESI) $[M + H]^+$: 521.3.

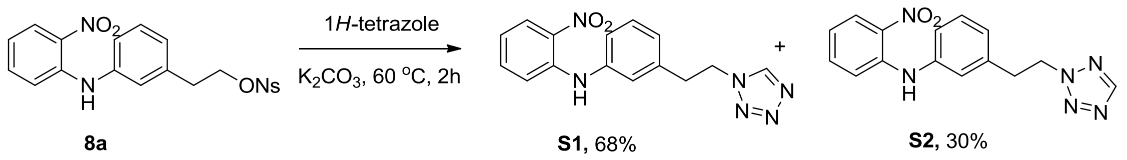

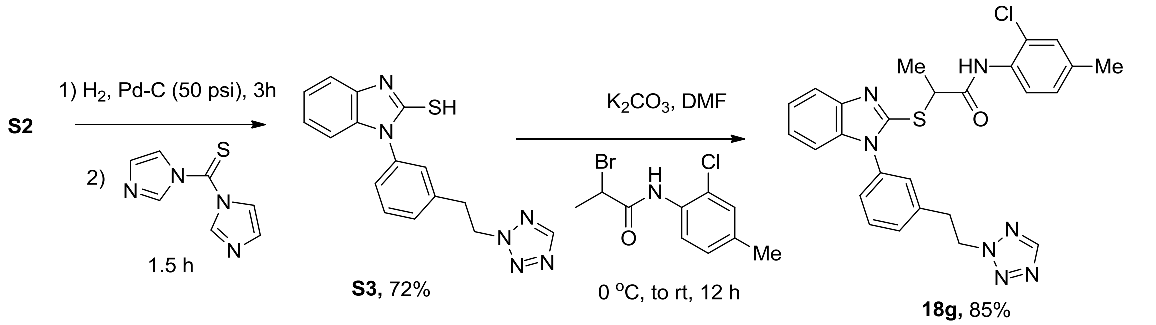

***N*-(3-(2-(1*H*-Tetrazol-1-yl)ethyl)phenyl)-2-nitroaniline (S1) and *N*-(3-(2-(2*H*-tetrazol-2-yl)ethyl)phenyl)-2-nitroaniline (S2):** To a stirred mixture of **8a** (250 mg, 0.564 mmol) and K_2CO_3 (95 mg, 0.677 mmol) in DMF (1.5 mL) at room temperature was added 1*H*-tetrazole (60 mg, 0.845 mmol). The reaction mixture was then heated to 60 ºC and stirred at that temperature for 2 h. Then the reaction mixture was concentrated, dissolved in ethyl acetate, and washed with water. The aqueous phase was extracted with ethyl acetate and the combined organic phase was washed with water and brine, dried over Na_2SO_4 and concentrated. Chromatographic purification using 15% EtOAc in hexanes afforded **S1** (128 mg, 68%), and eluting with 25% EtOAc in hexanes afforded **S2** (60 mg, 30%) as a reddish brown solid.

^{1}H NMR for **S1**: (300 MHz, $CDCl_3$) δ 9.39 (s, 1 H), 8.38 (s, 1H), 8.16 (d, *J* = 8.7 Hz, 1H), 7.40 (t, *J* = 7.8 Hz, 1H), 7.32 (t, *J* = 7.8 Hz, 1H), 7.15 (d, *J* = 7.8 Hz, 1H), 7.06 (d, *J* = 8.4 Hz, 1H), 6.99 (s, 1H), 6.88 (d, *J* = 7.2 Hz, 1H), 6.78 (t, *J* = 7.2 Hz, 1H), 4.72 (t, *J* = 6.6 Hz, 2H), 3.26 (t, *J* = 6.6 Hz, 2H). *m/z* (ESI) $[M + H]^+$: 311.1.

^{1}H NMR for **S2**: (300 MHz, $CDCl_3$) δ 9.39 (s, 1 H), 8.35 (s, 1H), 8.17 (d, *J* = 8.4 Hz, 1H), 7.41 (t, *J* = 7.8 Hz, 1H), 7.33 (t, *J* = 7.8 Hz, 1H), 7.16 (d, *J* = 8.1 Hz, 1H), 7.06 (d, *J* = 8.1 Hz, 1H), 6.99 (s, 1H), 6.89 (d, *J* = 7.2 Hz, 1H), 6.79 (t, *J* = 7.2 Hz, 1H), 4.71 (t, *J* = 6.6 Hz, 2H), 3.26 (t, *J* = 6.6 Hz, 2H). *m/z* (ESI) $[M + H]^+$: 311.1.

1-(3-(2-(2*H*-Tetrazol-2-yl)ethyl)phenyl)-1*H*-benzo[*d*]imidazole-2-thiol (S3): Following the same procedure as for the preparation of **9b**, using **S2** (80 mg, 0.258 mmol) as substrate, **S3** (60 mg, 72% over two steps) was obtained as colorless solid. ^{1}H NMR (300 MHz, $CDCl_3$) δ 8.49 (s, 1H), 7.50-7.45 (m, 2H), 7.39 (d, *J* = 7.8 Hz, 1H), 7.31 (t, *J* = 7.8 Hz, 1H), 7.19 (t, *J* = 7.8 Hz, 1H), 7.09 (d, *J* = 7.8 Hz, 1H), 6.92 (d, *J* = 7.8 Hz, 1H), 4.78 (t, *J* = 6.6 Hz, 2H), 3.34 (t, *J* = 6.6 Hz, 2H). *m*/*z* (ESI) $[M + H]^+$: 322.0.

2-((1-(3-(2-(2*H*-Tetrazol-2-yl)ethyl)phenyl)-1*H*-benzo[*d*]imidazol-2-yl)thio)-*N*-(2-chloro-4-methylphenyl)propanamide (18g): To a stirred mixture of **S3** (55 mg, 0.17 mmol) and potassium carbonate (25 mg, 0.18 mmol) in DMF (1.5 mL) at 0 °C was added 2-bromo-*N*-(2-chloro-4-methylphenyl)propanamide (50 mg, 0.18 mmol). After stirring at room temperature for 12 h, the mixture was diluted with EtOAc, and the organic layer was washed with water and brine, dried over Na_2SO_4, filtered and concentrated. Chromatographic purification of crude using 40–50% ethyl acetate in hexanes provided **18g** (75 mg, 85%) as a white solid. ^{1}H NMR (300 MHz, $CDCl_3$) δ 10.31 (s, 1H), 8.39 (s, 1H), 8.22 (d, *J* = 8.1 Hz, 1H), 7.73 (d, *J* = 8.1 Hz, 1H), 7.49 (t, *J* = 7.5 Hz, 1H), 7.37-7.17 (m, 4H), 7.12 (s, 1H), 7.08-7.04 (m, 2H), 4.96 (q, *J* = 7.2 Hz, 1H), 4.72 (t, *J* = 7.2 Hz, 2H), 3.34 (t, *J* = 7.2 Hz, 2H), 2.25 (s, 3H), 1.64 (d, *J* = 7.2 Hz, 3H). *m*/*z* (ESI) $[M + H]^+$: 518.2.

Formation of L-Tartaric acid salt of 18f. Compound **18f** (261 mg, 0.5 mmol) (12f) in and L-tartaric acid (75 mg, 0.5 mmol) were dissolved in 5 mL abs. ethanol to obtain clear solution. The mixture was allowed to stand at ambient temperature with trituration. The tartaric acid salt gradually precipitated out from the solution and cooled, and the solid was collected by filtration, washed with ethanol and hexanes, and dried at 40 °C for 18 h to obtain a dried solid as the tartaric acid salt.

Table 1: Crystal data and structure refinement for (*S*)-**1**.

Identification code	twm	
Empirical formula	C24 H22 Cl N3 O2 S	
Formula weight	451.96	
Temperature	173(2) K	
Wavelength	1.54184 Å	
Crystal system	Orthorhombic	
Space group	P 21 21 21	
Unit cell dimensions	a = 8.6095(2) Å	a= 90°.
	b = 12.0675(2) Å	b= 90°.
	c = 21.4444(4) Å	g = 90°.
Volume	2227.97(8) $Å^3$	
Z	4	
Density (calculated)	1.347 Mg/m^3	
Absorption coefficient	2.605 mm^{-1}	
F(000)	944	
Crystal size	0.632 × 0.579 × 0.502 mm^3	
Theta range for data collection	4.12 to 69.09°.	
Index ranges	-10<=h<=9, -14<=k<=13, -25<=l<=25	
Reflections collected	16286	
Independent reflections	3977 [R(int) = 0.0208]	
Completeness to theta = 69.09°	99.3 %	
Absorption correction	Numerical	
Max. and min. transmission	0.4745 and 0.3182	
Refinement method	Full-matrix least-squares on F^2	
Data / restraints / parameters	3977 / 0 / 299	
Goodness-of-fit on F^2	1.125	
Final R indices [I>2sigma(I)]	R1 = 0.0319, wR2 = 0.0843	
R indices (all data)	R1 = 0.0320, wR2 = 0.0844	
Absolute structure parameter	0.041(12)	
Largest diff. peak and hole	0.607 and -0.249 e.$Å^{-3}$	

Table 2: Atomic coordinates (× 10^4) and equivalent isotropic displacement parameters ($Å^2$ × 10^3) for (*S*)-**1**. U(eq) is defined as one third of the trace of the orthogonalized U^{ij} tensor.

	x	y	z	U(eq)
C(1)	8554(2)	8656(2)	2885(1)	30(1)
C(2)	8401(2)	9715(2)	2648(1)	36(1)
C(3)	7791(2)	9893(2)	2054(1)	36(1)
C(4)	7398(2)	8967(2)	1708(1)	36(1)
C(5)	7514(2)	7902(2)	1951(1)	35(1)
C(6)	8077(2)	7720(1)	2550(1)	29(1)
C(7)	7575(3)	11044(2)	1797(1)	48(1)
C(8)	7544(3)	5710(2)	2590(1)	36(1)
C(9)	7748(2)	4692(2)	3011(1)	34(1)
C(10)	7377(3)	3621(2)	2656(1)	45(1)
C(11)	7606(2)	5340(1)	4231(1)	28(1)
C(12)	9409(2)	6209(1)	4717(1)	28(1)
C(13)	10691(2)	6848(2)	4889(1)	33(1)
C(14)	10916(2)	7036(2)	5520(1)	37(1)
C(15)	9908(3)	6610(2)	5970(1)	39(1)
C(16)	8620(2)	5981(2)	5812(1)	35(1)
C(17)	8394(2)	5799(1)	5178(1)	29(1)
C(18)	5941(2)	4657(2)	5112(1)	30(1)
C(19)	6196(3)	3749(2)	5493(1)	55(1)
C(20)	4957(3)	3192(2)	5749(2)	64(1)
C(21)	3451(3)	3537(2)	5630(1)	43(1)
C(22)	3193(2)	4440(2)	5248(1)	35(1)
C(23)	4449(2)	5005(1)	4995(1)	32(1)
C(24)	750(3)	3286(3)	5845(2)	80(1)
Cl(1)	9395(1)	8498(1)	3618(1)	40(1)
N(1)	8183(2)	6659(1)	2819(1)	31(1)
N(2)	8880(2)	5906(1)	4128(1)	29(1)
N(3)	7246(2)	5237(1)	4852(1)	30(1)
O(1)	6903(3)	5632(1)	2094(1)	66(1)
O(2)	2336(2)	2933(2)	5915(1)	65(1)
S(1)	6395(1)	4764(1)	3668(1)	32(1)

Table 3: Bond lengths [Å] and angles [°] for (*S*)-**1**.

Cl(1)-C(1)	1.7405(18)
S(1)-C(9)	1.8301(18)
S(1)-C(11)	1.7396(17)
O(1)-C(8)	1.203(3)
O(2)-C(21)	1.350(3)
O(2)-C(24)	1.439(3)
N(1)-C(6)	1.408(2)
N(1)-C(8)	1.362(2)
N(2)-C(11)	1.311(2)
N(2)-C(12)	1.392(2)
N(3)-C(11)	1.374(2)
N(3)-C(17)	1.387(2)
N(3)-C(18)	1.437(2)
N(1)-H(1)	0.8800
C(1)-C(6)	1.400(2)
C(1)-C(2)	1.381(3)
C(2)-C(3)	1.395(3)
C(3)-C(7)	1.506(3)
C(3)-C(4)	1.383(3)
C(4)-C(5)	1.390(3)
C(5)-C(6)	1.391(3)
C(8)-C(9)	1.533(3)
C(9)-C(10)	1.534(3)
C(12)-C(13)	1.396(2)
C(12)-C(17)	1.408(2)
C(13)-C(14)	1.386(3)
C(14)-C(15)	1.395(3)
C(15)-C(16)	1.386(3)
C(16)-C(17)	1.392(2)
C(18)-C(23)	1.375(2)
C(18)-C(19)	1.384(3)
C(19)-C(20)	1.375(4)
C(20)-C(21)	1.386(4)
C(21)-C(22)	1.381(3)

C(22)-C(23)	1.389(2)
C(2)-H(2)	0.9500
C(4)-H(4)	0.9500
C(5)-H(5)	0.9500
C(7)-H(7A)	0.9800
C(7)-H(7B)	0.9800
C(7)-H(7C)	0.9800
C(9)-H(9)	1.0000
C(10)-H(10A)	0.9800
C(10)-H(10B)	0.9800
C(10)-H(10C)	0.9800
C(13)-H(13)	0.9500
C(14)-H(14)	0.9500
C(15)-H(15)	0.9500
C(16)-H(16)	0.9500
C(19)-H(19)	0.9500
C(20)-H(20)	0.9500
C(22)-H(22)	0.9500
C(23)-H(23)	0.9500
C(24)-H(24A)	0.9800
C(24)-H(24B)	0.9800
C(24)-H(24C)	0.9800
C(9)-S(1)-C(11)	99.89(8)
C(21)-O(2)-C(24)	117.9(2)
C(6)-N(1)-C(8)	126.25(16)
C(11)-N(2)-C(12)	104.90(14)
C(11)-N(3)-C(17)	106.45(14)
C(11)-N(3)-C(18)	126.69(14)
C(17)-N(3)-C(18)	126.86(15)
C(6)-N(1)-H(1)	117.00
C(8)-N(1)-H(1)	117.00
C(2)-C(1)-C(6)	121.95(16)
Cl(1)-C(1)-C(2)	118.26(14)
Cl(1)-C(1)-C(6)	119.78(14)
C(1)-C(2)-C(3)	121.00(17)

C(2)-C(3)-C(7)	121.51(18)
C(4)-C(3)-C(7)	121.29(19)
C(2)-C(3)-C(4)	117.20(18)
C(3)-C(4)-C(5)	121.89(18)
C(4)-C(5)-C(6)	121.15(17)
N(1)-C(6)-C(1)	120.31(15)
C(1)-C(6)-C(5)	116.66(16)
N(1)-C(6)-C(5)	123.03(16)
N(1)-C(8)-C(9)	114.57(17)
O(1)-C(8)-C(9)	120.68(18)
O(1)-C(8)-N(1)	124.72(18)
S(1)-C(9)-C(8)	109.96(13)
S(1)-C(9)-C(10)	106.83(13)
C(8)-C(9)-C(10)	111.08(15)
S(1)-C(11)-N(2)	126.34(13)
N(2)-C(11)-N(3)	113.54(15)
S(1)-C(11)-N(3)	120.11(12)
N(2)-C(12)-C(17)	109.98(14)
N(2)-C(12)-C(13)	130.04(16)
C(13)-C(12)-C(17)	119.94(16)
C(12)-C(13)-C(14)	117.36(16)
C(13)-C(14)-C(15)	121.80(17)
C(14)-C(15)-C(16)	122.10(18)
C(15)-C(16)-C(17)	115.87(16)
N(3)-C(17)-C(16)	131.95(16)
C(12)-C(17)-C(16)	122.92(16)
N(3)-C(17)-C(12)	105.13(14)
C(19)-C(18)-C(23)	119.88(18)
N(3)-C(18)-C(19)	119.39(17)
N(3)-C(18)-C(23)	120.73(16)
C(18)-C(19)-C(20)	120.0(2)
C(19)-C(20)-C(21)	120.4(2)
O(2)-C(21)-C(22)	125.4(2)
O(2)-C(21)-C(20)	114.8(2)
C(20)-C(21)-C(22)	119.8(2)
C(21)-C(22)-C(23)	119.61(18)

C(18)-C(23)-C(22)	120.38(16)
C(1)-C(2)-H(2)	120.00
C(3)-C(2)-H(2)	119.00
C(3)-C(4)-H(4)	119.00
C(5)-C(4)-H(4)	119.00
C(4)-C(5)-H(5)	119.00
C(6)-C(5)-H(5)	119.00
C(3)-C(7)-H(7A)	110.00
C(3)-C(7)-H(7B)	109.00
C(3)-C(7)-H(7C)	109.00
H(7A)-C(7)-H(7B)	109.00
H(7A)-C(7)-H(7C)	109.00
H(7B)-C(7)-H(7C)	109.00
S(1)-C(9)-H(9)	110.00
C(8)-C(9)-H(9)	110.00
C(10)-C(9)-H(9)	110.00
C(9)-C(10)-H(10A)	109.00
C(9)-C(10)-H(10B)	109.00
C(9)-C(10)-H(10C)	109.00
H(10A)-C(10)-H(10B)	109.00
H(10A)-C(10)-H(10C)	109.00
H(10B)-C(10)-H(10C)	110.00
C(12)-C(13)-H(13)	121.00
C(14)-C(13)-H(13)	121.00
C(13)-C(14)-H(14)	119.00
C(15)-C(14)-H(14)	119.00
C(14)-C(15)-H(15)	119.00
C(16)-C(15)-H(15)	119.00
C(15)-C(16)-H(16)	122.00
C(17)-C(16)-H(16)	122.00
C(18)-C(19)-H(19)	120.00
C(20)-C(19)-H(19)	120.00
C(19)-C(20)-H(20)	120.00
C(21)-C(20)-H(20)	120.00
C(21)-C(22)-H(22)	120.00
C(23)-C(22)-H(22)	120.00

C(18)-C(23)-H(23)	120.00
C(22)-C(23)-H(23)	120.00
O(2)-C(24)-H(24A)	109.00
O(2)-C(24)-H(24B)	109.00
O(2)-C(24)-H(24C)	109.00
H(24A)-C(24)-H(24B)	109.00
H(24A)-C(24)-H(24C)	110.00
H(24B)-C(24)-H(24C)	109.00

Symmetry transformations used to generate equivalent atoms:

Table 4: Anisotropic displacement parameters ($Å^2 \times 10^3$) for (*S*)-**1**. The anisotropic displacement factor exponent takes the form: $-2p^2 [h^2 a^{*2}U^{11} + ... + 2 h k a^* b^* U^{12}]$

	U^{11}	U^{22}	U^{33}	U^{23}	U^{13}	U^{12}
C(1)	30(1)	34(1)	27(1)	-3(1)	4(1)	-3(1)
C(2)	37(1)	32(1)	39(1)	-6(1)	5(1)	-3(1)
C(3)	34(1)	32(1)	43(1)	3(1)	4(1)	1(1)
C(4)	34(1)	39(1)	34(1)	4(1)	-4(1)	2(1)
C(5)	40(1)	31(1)	34(1)	-2(1)	-7(1)	0(1)
C(6)	30(1)	30(1)	29(1)	-3(1)	3(1)	0(1)
C(7)	55(1)	33(1)	56(1)	8(1)	-2(1)	1(1)
C(8)	50(1)	31(1)	27(1)	-3(1)	0(1)	-4(1)
C(9)	43(1)	29(1)	28(1)	-5(1)	4(1)	-3(1)
C(10)	67(1)	30(1)	37(1)	-8(1)	6(1)	-5(1)
C(11)	30(1)	26(1)	27(1)	1(1)	-1(1)	-1(1)
C(12)	29(1)	27(1)	29(1)	2(1)	-1(1)	2(1)
C(13)	31(1)	30(1)	37(1)	4(1)	-2(1)	-1(1)
C(14)	37(1)	30(1)	43(1)	0(1)	-11(1)	-2(1)
C(15)	47(1)	42(1)	29(1)	-1(1)	-10(1)	1(1)
C(16)	38(1)	40(1)	28(1)	2(1)	-1(1)	0(1)
C(17)	30(1)	28(1)	29(1)	2(1)	-2(1)	1(1)
C(18)	30(1)	32(1)	29(1)	2(1)	2(1)	-4(1)
C(19)	29(1)	63(1)	73(2)	37(1)	-1(1)	0(1)
C(20)	40(1)	68(2)	86(2)	50(2)	1(1)	-1(1)
C(21)	35(1)	42(1)	53(1)	12(1)	6(1)	-4(1)
C(22)	28(1)	33(1)	44(1)	2(1)	1(1)	1(1)
C(23)	34(1)	30(1)	32(1)	2(1)	0(1)	-1(1)
C(24)	34(1)	72(2)	132(3)	42(2)	27(2)	4(1)
Cl(1)	55(1)	38(1)	26(1)	-4(1)	-3(1)	-11(1)
N(1)	38(1)	30(1)	25(1)	-1(1)	-2(1)	-2(1)
N(2)	29(1)	29(1)	28(1)	1(1)	1(1)	-3(1)
N(3)	30(1)	32(1)	26(1)	3(1)	0(1)	-3(1)
O(1)	120(2)	40(1)	38(1)	2(1)	-30(1)	-23(1)
O(2)	34(1)	62(1)	97(2)	39(1)	14(1)	-1(1)
S(1)	33(1)	36(1)	29(1)	-4(1)	-1(1)	-7(1)

Table 5: Hydrogen coordinates (× 10^4) and isotropic displacement parameters ($Å^2$ × 10^3) for (*S*)-**1**.

	x	y	z	U(eq)
H(2)	8716	10331	2893	53(7)
H(4)	7037	9061	1292	38(6)
H(5)	7204	7288	1703	44(7)
H(7A)	7617	11021	1341	72
H(7B)	8402	11527	1954	72
H(7C)	6564	11334	1930	72
H(9)	8839	4664	3170	33(5)
H(10A)	6283	3626	2529	50(7)
H(10B)	7570	2982	2927	59(8)
H(10C)	8039	3571	2285	51(7)
H(13)	11383	7141	4586	59(8)
H(14)	11780	7468	5650	52(7)
H(15)	10111	6756	6397	22(4)
H(16)	7934	5691	6118	29(5)
H(19)	7226	3511	5577	65(9)
H(20)	5135	2567	6009	58(8)
H(22)	2163	4672	5159	36(6)
H(23)	4276	5636	4739	41(6)
H(24A)	467	3277	5402	119
H(24B)	635	4039	6009	119
H(24C)	68	2782	6076	119
H(1)	8714	6603	3168	37(6)

Table 6: Torsion angles [°] for (*S*)-**1**.

C(11)-S(1)-C(9)-C(10)	143.27(13)
C(9)-S(1)-C(11)-N(2)	21.36(16)
C(9)-S(1)-C(11)-N(3)	-159.82(13)
C(11)-S(1)-C(9)-C(8)	-96.09(14)
C(24)-O(2)-C(21)-C(22)	-3.6(4)
C(24)-O(2)-C(21)-C(20)	176.1(3)
C(6)-N(1)-C(8)-C(9)	-175.51(17)
C(8)-N(1)-C(6)-C(5)	-11.4(3)
C(6)-N(1)-C(8)-O(1)	6.6(4)
C(8)-N(1)-C(6)-C(1)	168.87(19)
C(12)-N(2)-C(11)-N(3)	-0.30(18)
C(12)-N(2)-C(11)-S(1)	178.59(12)
C(11)-N(2)-C(12)-C(13)	-177.86(18)
C(11)-N(2)-C(12)-C(17)	-0.05(18)
C(17)-N(3)-C(18)-C(23)	116.0(2)
C(11)-N(3)-C(18)-C(23)	-64.2(2)
C(17)-N(3)-C(18)-C(19)	-63.0(3)
C(18)-N(3)-C(17)-C(16)	-0.9(3)
C(17)-N(3)-C(11)-S(1)	-178.44(12)
C(11)-N(3)-C(18)-C(19)	116.8(2)
C(18)-N(3)-C(11)-N(2)	-179.27(15)
C(17)-N(3)-C(11)-N(2)	0.53(19)
C(18)-N(3)-C(17)-C(12)	179.29(15)
C(18)-N(3)-C(11)-S(1)	1.8(2)
C(11)-N(3)-C(17)-C(16)	179.28(18)
C(11)-N(3)-C(17)-C(12)	-0.51(17)
C(2)-C(1)-C(6)-N(1)	-176.97(16)
C(2)-C(1)-C(6)-C(5)	3.3(3)
Cl(1)-C(1)-C(6)-C(5)	-175.70(13)
C(6)-C(1)-C(2)-C(3)	-1.5(3)
Cl(1)-C(1)-C(2)-C(3)	177.54(14)
Cl(1)-C(1)-C(6)-N(1)	4.1(2)
C(1)-C(2)-C(3)-C(4)	-2.1(3)
C(1)-C(2)-C(3)-C(7)	178.19(18)

C(7)-C(3)-C(4)-C(5)	-176.53(18)
C(2)-C(3)-C(4)-C(5)	3.7(3)
C(3)-C(4)-C(5)-C(6)	-1.9(3)
C(4)-C(5)-C(6)-C(1)	-1.6(3)
C(4)-C(5)-C(6)-N(1)	178.62(17)
N(1)-C(8)-C(9)-S(1)	76.4(2)
O(1)-C(8)-C(9)-C(10)	12.3(3)
N(1)-C(8)-C(9)-C(10)	-165.60(18)
O(1)-C(8)-C(9)-S(1)	-105.7(2)
N(2)-C(12)-C(13)-C(14)	178.55(17)
C(13)-C(12)-C(17)-N(3)	178.42(15)
C(17)-C(12)-C(13)-C(14)	0.9(2)
N(2)-C(12)-C(17)-N(3)	0.36(18)
N(2)-C(12)-C(17)-C(16)	-179.46(15)
C(13)-C(12)-C(17)-C(16)	-1.4(3)
C(12)-C(13)-C(14)-C(15)	-0.1(3)
C(13)-C(14)-C(15)-C(16)	-0.5(3)
C(14)-C(15)-C(16)-C(17)	0.0(3)
C(15)-C(16)-C(17)-N(3)	-178.89(18)
C(15)-C(16)-C(17)-C(12)	0.9(3)
N(3)-C(18)-C(23)-C(22)	179.97(17)
C(19)-C(18)-C(23)-C(22)	-1.1(3)
N(3)-C(18)-C(19)-C(20)	179.5(2)
C(23)-C(18)-C(19)-C(20)	0.5(3)
C(18)-C(19)-C(20)-C(21)	-0.3(4)
C(19)-C(20)-C(21)-C(22)	0.6(4)
C(19)-C(20)-C(21)-O(2)	-179.2(2)
O(2)-C(21)-C(22)-C(23)	178.6(2)
C(20)-C(21)-C(22)-C(23)	-1.1(3)
C(21)-C(22)-C(23)-C(18)	1.4(3)

Symmetry transformations used to generate equivalent atoms:

Table 7: Hydrogen bonds for (*S*)-**1** [Å and °].

D-H...A	d(D-H)	d(H...A)	d(D...A)	<(DHA)
N(1)-H(1)...Cl(1)	0.8800	2.5500	2.9914(16)	112.00
N(1)-H(1)...N(2)	0.8800	2.2300	3.010(2)	148.00
C(5)-H(5)...O(1)	0.9500	2.1800	2.807(3)	122.00
C(9)-H(9)...N(2)	1.0000	2.5400	2.972(2)	106.00

Symmetry transformations used to generate equivalent atoms:

Table 8: Crystal data and structure refinement for (*R*)-**1**.

Identification code	twin4	
Empirical formula	C24 H22 Cl N3 O2 S	
Formula weight	451.96	
Temperature	173 K	
Wavelength	1.54184 Å	
Crystal system	Orthorhombic	
Space group	P 21 21 21	
Unit cell dimensions	a = 8.6201(3) Å	□= 90°.
	b = 12.0676(3) Å	□= 90°.
	c = 21.4502(7) Å	□ = 90°.
Volume	2231.33(12) $Å^3$	
Z	4	
Density (calculated)	1.345 Mg/m^3	
Absorption coefficient	2.601 mm^{-1}	
F(000)	944	
Crystal size	0.333 x 0.214 x 0.175 mm^3	
Theta range for data collection	4.12 to 69.03°.	
Index ranges	-10<=h<=10, -14<=k<=14, -25<=l<=25	
Reflections collected	30852	
Independent reflections	4098 [R(int) = 0.0467]	
Completeness to theta = 69.03°	99.2 %	
Absorption correction	Semi-empirical from equivalents	
Max. and min. transmission	0.7531 and 0.5536	
Refinement method	Full-matrix least-squares on F^2	
Data / restraints / parameters	4098 / 0 / 298	
Goodness-of-fit on F^2	1.027	
Final R indices [I>2sigma(I)]	R1 = 0.0387, wR2 = 0.1024	
R indices (all data)	R1 = 0.0405, wR2 = 0.1037	
Absolute structure parameter	0.007(14)	
Largest diff. peak and hole	0.477 and -0.244 e.$Å^{-3}$	

Table 9: Atomic coordinates (× 10^4) and equivalent isotropic displacement parameters ($Å^2$ × 10^3) for (*R*)-**1**. U(eq) is defined as one third of the trace of the orthogonalized U^{ij} tensor.

	x	y	z	U(eq)
Cl(30)	5615(1)	1499(1)	1383(1)	44(1)
S(16)	8610(1)	5233(1)	1332(1)	37(1)
O(19)	12664(2)	7070(2)	-918(1)	71(1)
O(22)	8105(3)	4363(2)	2905(1)	68(1)
N(1)	7760(2)	4762(2)	149(1)	34(1)
N(3)	6122(2)	4093(1)	874(1)	34(1)
N(23)	6818(2)	3340(2)	2181(1)	35(1)
C(2)	7398(2)	4662(2)	769(1)	32(1)
C(4)	5601(2)	3794(2)	284(1)	33(1)
C(5)	6606(3)	4199(2)	-179(1)	34(1)
C(6)	6384(3)	4018(2)	-811(1)	41(1)
C(7)	5094(3)	3391(2)	-970(1)	43(1)
C(8)	4089(3)	2963(2)	-519(1)	41(1)
C(9)	4316(3)	3157(2)	109(1)	37(1)
C(10)	9056(2)	5347(2)	-115(1)	35(1)
C(11)	10550(3)	4991(2)	4(1)	36(1)
C(12)	11806(3)	5562(2)	-251(1)	41(1)
C(13)	11550(3)	6463(2)	-629(1)	47(1)
C(14)	10030(3)	6804(3)	-749(2)	70(1)
C(15)	8808(3)	6250(3)	-494(2)	60(1)
C(17)	7254(3)	5308(2)	1989(1)	37(1)
C(18)	7616(4)	6381(2)	2342(1)	49(1)
C(20)	14244(4)	6714(3)	-845(2)	84(2)
C(21)	7453(3)	4293(2)	2410(1)	41(1)
C(24)	6926(3)	2280(2)	2449(1)	33(1)
C(25)	7489(3)	2099(2)	3050(1)	39(1)
C(26)	7604(3)	1034(2)	3293(1)	41(1)
C(27)	7202(3)	106(2)	2949(1)	42(1)
C(28)	6605(3)	285(2)	2355(1)	40(1)
C(29)	6452(3)	1340(2)	2118(1)	35(1)
C(31)	7424(4)	-1041(2)	3207(2)	53(1)

Table 10: Bond lengths [Å] and angles [°] for (*R*)-**1**.

Cl(30)-C(29)	1.745(2)
S(16)-C(2)	1.741(2)
S(16)-C(17)	1.832(3)
O(19)-C(13)	1.358(3)
O(19)-C(20)	1.437(4)
O(22)-C(21)	1.205(3)
N(1)-C(2)	1.371(3)
N(1)-C(5)	1.394(3)
N(1)-C(10)	1.437(3)
N(3)-C(2)	1.316(3)
N(3)-C(4)	1.391(3)
N(23)-C(21)	1.365(3)
N(23)-C(24)	1.407(3)
N(23)-H(23)	0.89(3)
C(4)-C(9)	1.400(3)
C(4)-C(5)	1.405(3)
C(5)-C(6)	1.388(3)
C(6)-C(7)	1.387(4)
C(7)-C(8)	1.397(4)
C(8)-C(9)	1.382(4)
C(10)-C(15)	1.378(4)
C(10)-C(11)	1.381(3)
C(11)-C(12)	1.395(4)
C(12)-C(13)	1.374(4)
C(13)-C(14)	1.397(4)
C(14)-C(15)	1.362(4)
C(17)-C(18)	1.532(4)
C(17)-C(21)	1.531(3)
C(24)-C(29)	1.399(3)
C(24)-C(25)	1.394(4)
C(25)-C(26)	1.391(3)
C(26)-C(27)	1.386(4)
C(27)-C(31)	1.503(4)
C(27)-C(28)	1.391(4)

C(28)-C(29)	1.378(3)
C(6)-H(6)	0.9500
C(7)-H(7)	0.9500
C(8)-H(8)	0.9500
C(9)-H(9)	0.9500
C(11)-H(11)	0.9500
C(12)-H(12)	0.9500
C(14)-H(14)	0.9500
C(15)-H(15)	0.9500
C(17)-H(17)	1.0000
C(18)-H(18A)	0.9800
C(18)-H(18B)	0.9800
C(18)-H(18C)	0.9800
C(20)-H(20A)	0.9800
C(20)-H(20B)	0.9800
C(20)-H(20C)	0.9800
C(25)-H(25)	0.9500
C(26)-H(26)	0.9500
C(28)-H(28)	0.9500
C(31)-H(31A)	0.9800
C(31)-H(31B)	0.9800
C(31)-H(31C)	0.9800

C(2)-S(16)-C(17)	99.82(11)
C(13)-O(19)-C(20)	117.3(3)
C(2)-N(1)-C(5)	106.45(17)
C(2)-N(1)-C(10)	126.99(18)
C(5)-N(1)-C(10)	126.56(19)
C(2)-N(3)-C(4)	104.42(18)
C(21)-N(23)-C(24)	126.4(2)
C(21)-N(23)-H(23)	108.2(16)
C(24)-N(23)-H(23)	124.3(16)
N(1)-C(2)-N(3)	113.77(19)
S(16)-C(2)-N(1)	120.14(14)
S(16)-C(2)-N(3)	126.07(18)
C(5)-C(4)-C(9)	119.4(2)

N(3)-C(4)-C(5)	110.66(17)
N(3)-C(4)-C(9)	129.9(2)
N(1)-C(5)-C(4)	104.71(19)
N(1)-C(5)-C(6)	131.9(2)
C(4)-C(5)-C(6)	123.4(2)
C(5)-C(6)-C(7)	115.8(2)
C(6)-C(7)-C(8)	122.0(2)
C(7)-C(8)-C(9)	121.7(2)
C(4)-C(9)-C(8)	117.8(2)
N(1)-C(10)-C(15)	120.03(18)
C(11)-C(10)-C(15)	120.0(2)
N(1)-C(10)-C(11)	120.0(2)
C(10)-C(11)-C(12)	119.9(2)
C(11)-C(12)-C(13)	119.8(2)
O(19)-C(13)-C(12)	125.7(2)
C(12)-C(13)-C(14)	119.5(3)
O(19)-C(13)-C(14)	114.8(3)
C(13)-C(14)-C(15)	120.4(3)
C(10)-C(15)-C(14)	120.4(3)
C(18)-C(17)-C(21)	111.2(2)
S(16)-C(17)-C(18)	106.98(18)
S(16)-C(17)-C(21)	109.99(17)
N(23)-C(21)-C(17)	114.7(2)
O(22)-C(21)-C(17)	121.1(2)
O(22)-C(21)-N(23)	124.3(2)
N(23)-C(24)-C(29)	120.7(2)
N(23)-C(24)-C(25)	122.9(2)
C(25)-C(24)-C(29)	116.4(2)
C(24)-C(25)-C(26)	121.1(2)
C(25)-C(26)-C(27)	121.9(3)
C(28)-C(27)-C(31)	121.9(2)
C(26)-C(27)-C(28)	117.1(2)
C(26)-C(27)-C(31)	121.1(3)
C(27)-C(28)-C(29)	121.2(2)
C(24)-C(29)-C(28)	122.3(2)
Cl(30)-C(29)-C(24)	119.38(17)

Cl(30)-C(29)-C(28)	118.37(18)
C(5)-C(6)-H(6)	122.00
C(7)-C(6)-H(6)	122.00
C(6)-C(7)-H(7)	119.00
C(8)-C(7)-H(7)	119.00
C(7)-C(8)-H(8)	119.00
C(9)-C(8)-H(8)	119.00
C(4)-C(9)-H(9)	121.00
C(8)-C(9)-H(9)	121.00
C(10)-C(11)-H(11)	120.00
C(12)-C(11)-H(11)	120.00
C(11)-C(12)-H(12)	120.00
C(13)-C(12)-H(12)	120.00
C(13)-C(14)-H(14)	120.00
C(15)-C(14)-H(14)	120.00
C(10)-C(15)-H(15)	120.00
C(14)-C(15)-H(15)	120.00
S(16)-C(17)-H(17)	110.00
C(18)-C(17)-H(17)	110.00
C(21)-C(17)-H(17)	110.00
C(17)-C(18)-H(18A)	109.00
C(17)-C(18)-H(18B)	109.00
C(17)-C(18)-H(18C)	109.00
H(18A)-C(18)-H(18B)	110.00
H(18A)-C(18)-H(18C)	109.00
H(18B)-C(18)-H(18C)	109.00
O(19)-C(20)-H(20A)	109.00
O(19)-C(20)-H(20B)	110.00
O(19)-C(20)-H(20C)	109.00
H(20A)-C(20)-H(20B)	110.00
H(20A)-C(20)-H(20C)	109.00
H(20B)-C(20)-H(20C)	109.00
C(24)-C(25)-H(25)	120.00
C(26)-C(25)-H(25)	119.00
C(25)-C(26)-H(26)	119.00
C(27)-C(26)-H(26)	119.00

C(27)-C(28)-H(28)	119.00
C(29)-C(28)-H(28)	119.00
C(27)-C(31)-H(31A)	109.00
C(27)-C(31)-H(31B)	109.00
C(27)-C(31)-H(31C)	110.00
H(31A)-C(31)-H(31B)	109.00
H(31A)-C(31)-H(31C)	109.00
H(31B)-C(31)-H(31C)	109.00

Table 11: Anisotropic displacement parameters ($Å^2 \times 10^3$)for (*R*)-**1**. The anisotropic displacement factor exponent takes the form: $-2\pi^2[h^2a^{*2}U^{11} + ... + 2 h k a^* b^* U^{12}]$

	U^{11}	U^{22}	U^{33}	U^{23}	U^{13}	U^{12}
Cl(30)	56(1)	45(1)	31(1)	-3(1)	-3(1)	-11(1)
S(16)	33(1)	44(1)	34(1)	-4(1)	0(1)	-7(1)
O(19)	33(1)	72(1)	108(2)	42(1)	16(1)	-2(1)
O(22)	113(2)	48(1)	42(1)	2(1)	-28(1)	-21(1)
N(1)	29(1)	41(1)	33(1)	2(1)	0(1)	-3(1)
N(3)	30(1)	38(1)	33(1)	0(1)	2(1)	-3(1)
N(23)	37(1)	39(1)	31(1)	-2(1)	-2(1)	-1(1)
C(2)	30(1)	33(1)	33(1)	-2(1)	-1(1)	0(1)
C(4)	30(1)	32(1)	37(1)	2(1)	-1(1)	2(1)
C(5)	31(1)	34(1)	35(1)	3(1)	-2(1)	1(1)
C(6)	39(1)	48(1)	36(1)	3(1)	-2(1)	1(1)
C(7)	47(1)	46(1)	37(1)	-1(1)	-12(1)	0(1)
C(8)	39(1)	39(1)	46(1)	1(1)	-8(1)	-3(1)
C(9)	31(1)	36(1)	45(1)	2(1)	-3(1)	-3(1)
C(10)	30(1)	42(1)	33(1)	2(1)	2(1)	-3(1)
C(11)	34(1)	36(1)	37(1)	1(1)	1(1)	-1(1)
C(12)	30(1)	42(1)	51(2)	1(1)	2(1)	0(1)
C(13)	32(1)	49(1)	60(2)	13(1)	8(1)	-4(1)
C(14)	37(1)	75(2)	97(3)	52(2)	3(2)	2(1)
C(15)	31(1)	70(2)	78(2)	36(2)	1(1)	2(1)
C(17)	39(1)	40(1)	33(1)	-6(1)	4(1)	-2(1)
C(18)	62(2)	39(1)	46(2)	-8(1)	7(1)	-6(1)
C(20)	34(1)	82(2)	136(4)	41(2)	26(2)	3(2)
C(21)	47(1)	41(1)	36(1)	-3(1)	1(1)	-4(1)
C(24)	30(1)	38(1)	32(1)	-3(1)	3(1)	-1(1)
C(25)	40(1)	39(1)	38(1)	-3(1)	-6(1)	0(1)
C(26)	34(1)	47(1)	44(1)	3(1)	-5(1)	2(1)
C(27)	34(1)	39(1)	52(2)	2(1)	5(1)	1(1)
C(28)	39(1)	39(1)	42(1)	-6(1)	6(1)	-3(1)
C(29)	33(1)	42(1)	31(1)	-3(1)	4(1)	-3(1)
C(31)	54(2)	43(1)	62(2)	7(1)	0(1)	0(1)

Table 12: Hydrogen coordinates (× 10^4) and isotropic displacement parameters (Å^2 × 10^3) for (*R*)-**1**.

	x	y	z	U(eq)
H(6)	7072	4306	-1117	40(7)
H(7)	4889	3247	-1397	49(8)
H(8)	3228	2528	-648	59(9)
H(9)	3623	2867	412	78(12)
H(11)	10722	4359	259	43
H(12)	12836	5328	-165	49
H(14)	9848	7426	-1011	84
H(15)	7778	6488	-579	72
H(17)	6166	5335	1828	36(7)
H(18A)	8715	6391	2459	73
H(18B)	6975	6420	2719	73
H(18C)	7390	7019	2075	73
H(20A)	14938	7255	-1040	126
H(20B)	14382	5990	-1043	126
H(20C)	14489	6656	-400	126
H(23)	6500(30)	3470(20)	1793(15)	40(7)
H(25)	7800	2713	3297	45(8)
H(26)	7967	940	3708	44(7)
H(28)	6298	-331	2109	53(8)
H(31A)	6567	-1516	3071	78(12)
H(31B)	7441	-1008	3664	110(16)
H(31C)	8407	-1346	3056	83(12)

Table 13: Torsion angles [°] for (*R*)-**1**.

C(17)-S(16)-C(2)-N(1)	159.94(17)
C(17)-S(16)-C(2)-N(3)	-21.7(2)
C(2)-S(16)-C(17)-C(18)	-142.83(18)
C(2)-S(16)-C(17)-C(21)	96.28(18)
C(20)-O(19)-C(13)-C(12)	2.8(5)
C(20)-O(19)-C(13)-C(14)	-176.0(3)
C(10)-N(1)-C(2)-S(16)	-2.6(3)
C(10)-N(1)-C(2)-N(3)	178.80(19)
C(2)-N(1)-C(5)-C(4)	0.3(2)
C(2)-N(1)-C(5)-C(6)	-179.3(2)
C(10)-N(1)-C(5)-C(4)	-178.78(19)
C(5)-N(1)-C(2)-S(16)	178.36(15)
C(5)-N(1)-C(2)-N(3)	-0.2(2)
C(2)-N(1)-C(10)-C(15)	-116.4(3)
C(5)-N(1)-C(10)-C(11)	-116.4(3)
C(5)-N(1)-C(10)-C(15)	62.5(3)
C(10)-N(1)-C(5)-C(6)	1.7(4)
C(2)-N(1)-C(10)-C(11)	64.8(3)
C(4)-N(3)-C(2)-S(16)	-178.39(15)
C(2)-N(3)-C(4)-C(9)	178.1(2)
C(4)-N(3)-C(2)-N(1)	0.1(2)
C(2)-N(3)-C(4)-C(5)	0.1(2)
C(21)-N(23)-C(24)-C(25)	11.4(4)
C(21)-N(23)-C(24)-C(29)	-169.1(2)
C(24)-N(23)-C(21)-O(22)	-5.8(4)
C(24)-N(23)-C(21)-C(17)	175.3(2)
C(9)-C(4)-C(5)-N(1)	-178.46(19)
N(3)-C(4)-C(5)-N(1)	-0.2(2)
N(3)-C(4)-C(5)-C(6)	179.4(2)
C(5)-C(4)-C(9)-C(8)	-0.6(3)
N(3)-C(4)-C(9)-C(8)	-178.4(2)
C(9)-C(4)-C(5)-C(6)	1.1(3)
C(4)-C(5)-C(6)-C(7)	-0.6(3)
N(1)-C(5)-C(6)-C(7)	178.8(2)

C(5)-C(6)-C(7)-C(8)	-0.4(4)
C(6)-C(7)-C(8)-C(9)	1.0(4)
C(7)-C(8)-C(9)-C(4)	-0.4(3)
N(1)-C(10)-C(15)-C(14)	-179.5(3)
C(11)-C(10)-C(15)-C(14)	-0.7(5)
N(1)-C(10)-C(11)-C(12)	-179.9(2)
C(15)-C(10)-C(11)-C(12)	1.3(4)
C(10)-C(11)-C(12)-C(13)	-1.2(4)
C(11)-C(12)-C(13)-C(14)	0.5(4)
C(11)-C(12)-C(13)-O(19)	-178.2(3)
O(19)-C(13)-C(14)-C(15)	178.9(3)
C(12)-C(13)-C(14)-C(15)	0.1(5)
C(13)-C(14)-C(15)-C(10)	0.0(5)
S(16)-C(17)-C(21)-O(22)	104.9(3)
S(16)-C(17)-C(21)-N(23)	-76.1(2)
C(18)-C(17)-C(21)-O(22)	-13.4(4)
C(18)-C(17)-C(21)-N(23)	165.6(2)
N(23)-C(24)-C(25)-C(26)	-178.8(2)
C(29)-C(24)-C(25)-C(26)	1.7(4)
N(23)-C(24)-C(29)-Cl(30)	-3.7(3)
N(23)-C(24)-C(29)-C(28)	177.1(2)
C(25)-C(24)-C(29)-Cl(30)	175.85(19)
C(25)-C(24)-C(29)-C(28)	-3.4(4)
C(24)-C(25)-C(26)-C(27)	1.5(4)
C(25)-C(26)-C(27)-C(28)	-3.0(4)
C(25)-C(26)-C(27)-C(31)	176.3(3)
C(26)-C(27)-C(28)-C(29)	1.3(4)
C(31)-C(27)-C(28)-C(29)	-178.0(3)
C(27)-C(28)-C(29)-Cl(30)	-177.3(2)
C(27)-C(28)-C(29)-C(24)	2.0(4)

Table 14: Hydrogen bonds for (*R*)-**1** [Å and °].

D-H...A	d(D-H)	d(H...A)	d(D...A)	<(DHA)
N(23)-H(23)...Cl(30)	0.89(3)	2.65(3)	2.990(2)	104.0(18)
N(23)-H(23)...N(3)	0.89(3)	2.14(3)	3.007(3)	167(2)
C(17)-H(17)...N(3)	1.0000	2.5400	2.970(3)	106.00
C(25)-H(25)...O(22)	0.9500	2.1800	2.801(3)	122.00

Nephelometry

Test compounds were dissolved in 100% dimethyl sulfoxide (DMSO) to obtain a final concentration of 30 mg/mL. The 30 mg/mL stock solution was serially diluted (concentration profile: 30, 20, 15, 10, 7.5, 5, 2.5, 1.25, 0.63, 0.31, and 0.15 mg/mL) in test tubes with 100% DMSO. The concentration profile was transferred to 96 well microplates (Costar black clear bottom) and serially diluted to a final DMSO (EMD) concentration of 1% and a final drug concentration of 300, 200, 150, 100, 75, 50, 25, 12, 6, 3 and 1.5 µg/mL with phosphate buffered saline, pH 7.4 (Sigma). The microplates were incubated for 90 minutes in the dark at ambient temperature. Laser nephelometry (NEPHELOstar, BMG Lab Technologies) was used to determine the point (concentration) at which the solute began to precipitate out of solution and expressed as counts of refractive nephelometry units (RNU). Laser nephelometry is the measurement of forward scattered light when a laser beam is directed through a solution.

^{1}H NMR of (*R*)-**1**

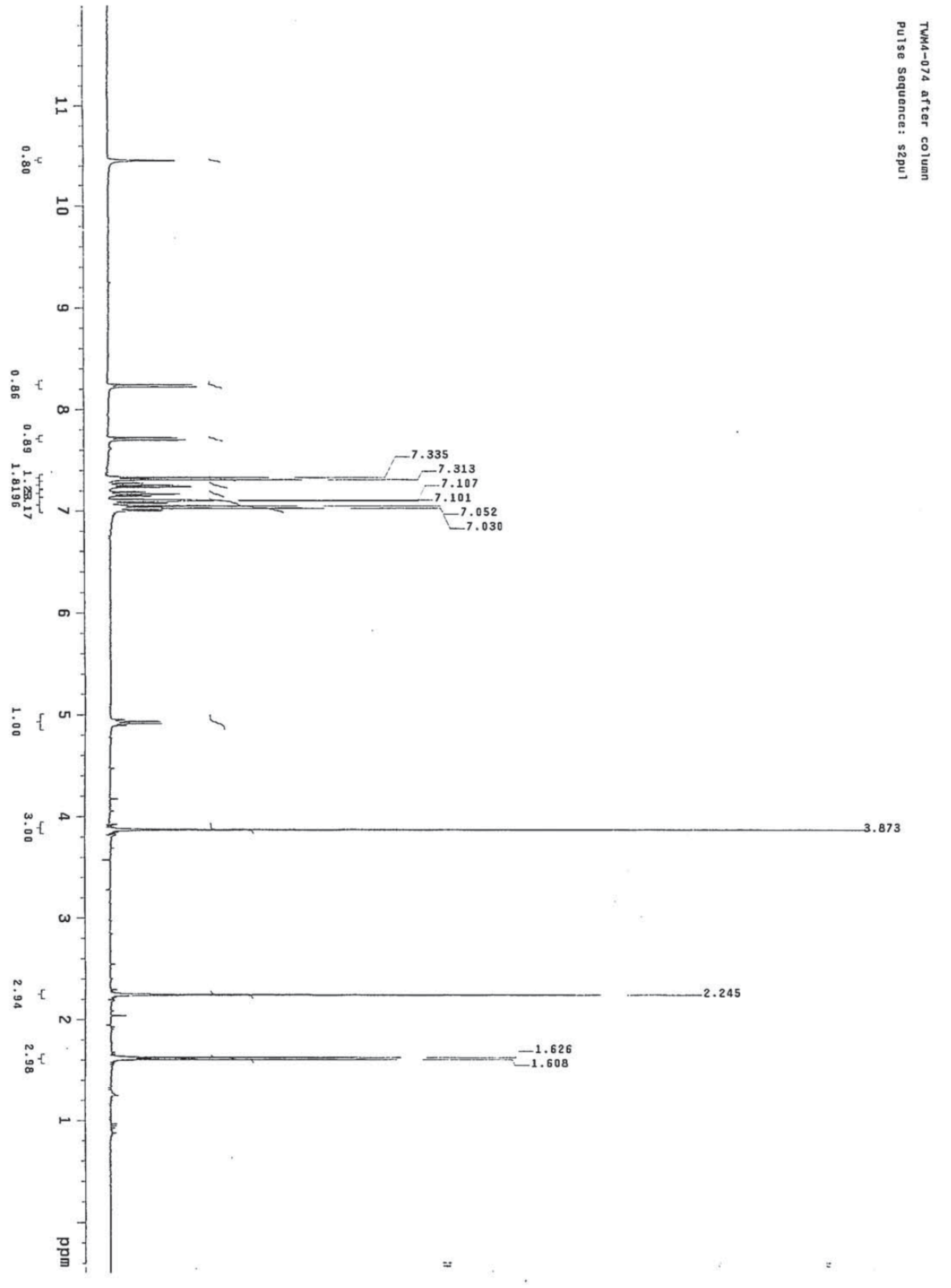
TWM4-074 after column
Pulse Sequence: s2pul
7.335
7.313
7.107
7.101
7.052
7.030
3.873
2.245
1.626
1.608
ppm

^{1}H NMR of (*S*)-**1**

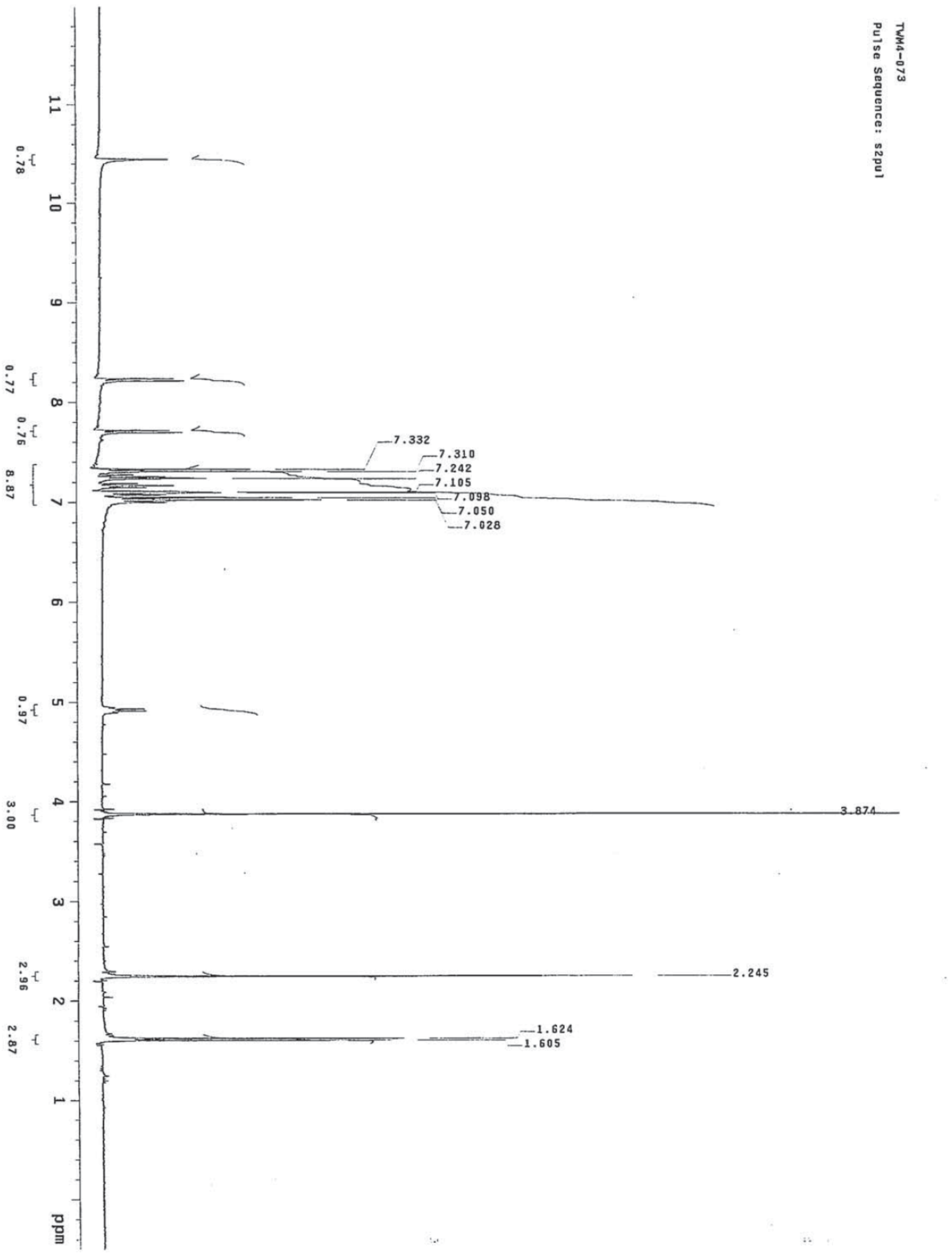

TWM4-073
Pulse Sequence: s2pul
7.332
7.310
7.242
7.105
7.098
7.050
7.028
3.874
2.245
1.624
1.605
0.78
0.77
0.76
8.87
0.97
3.00
2.96
2.87
ppm

^{1}H NMR of (*R*)-**14a**

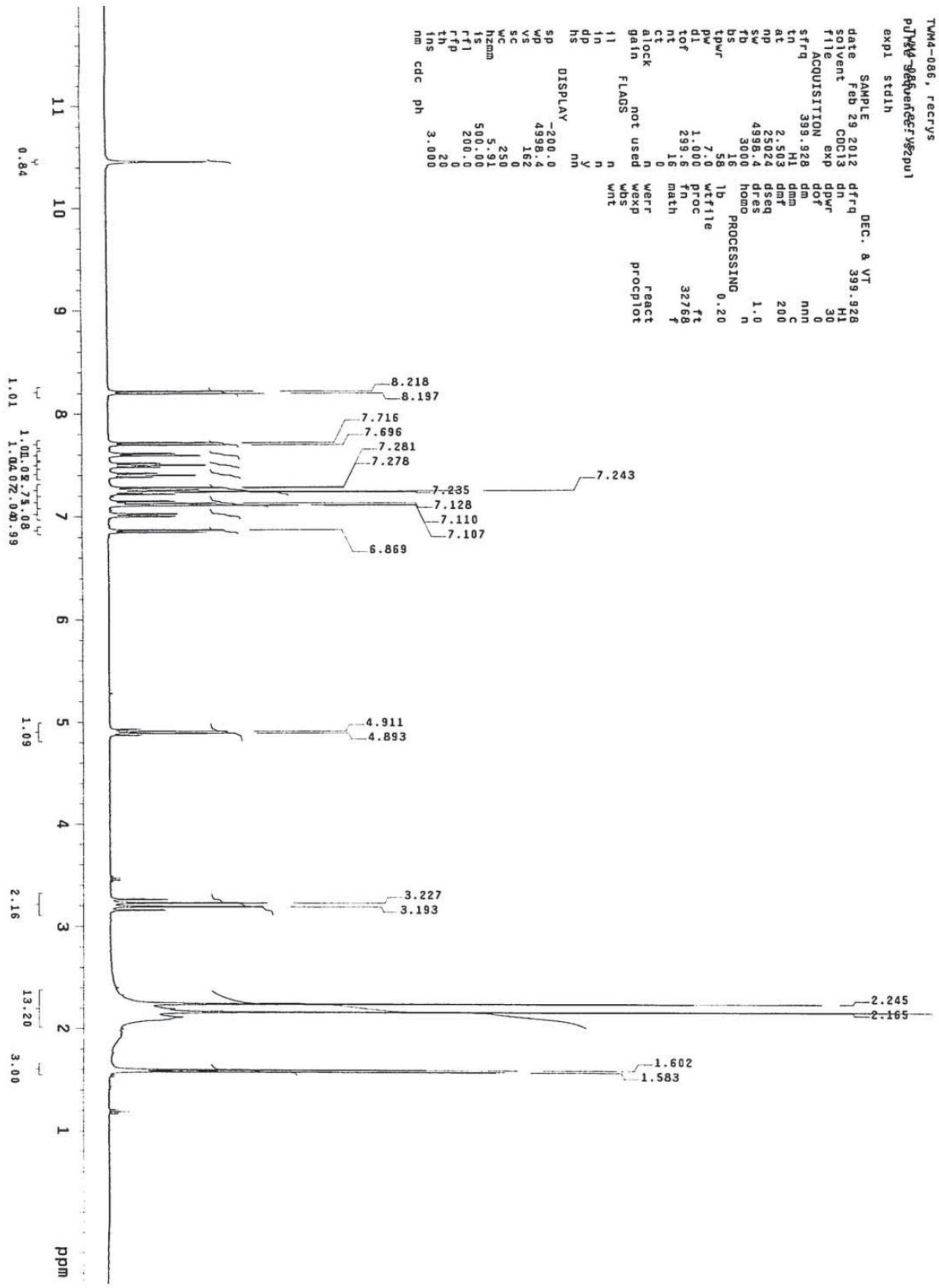

^{1}H NMR of (*S*)-**14a**

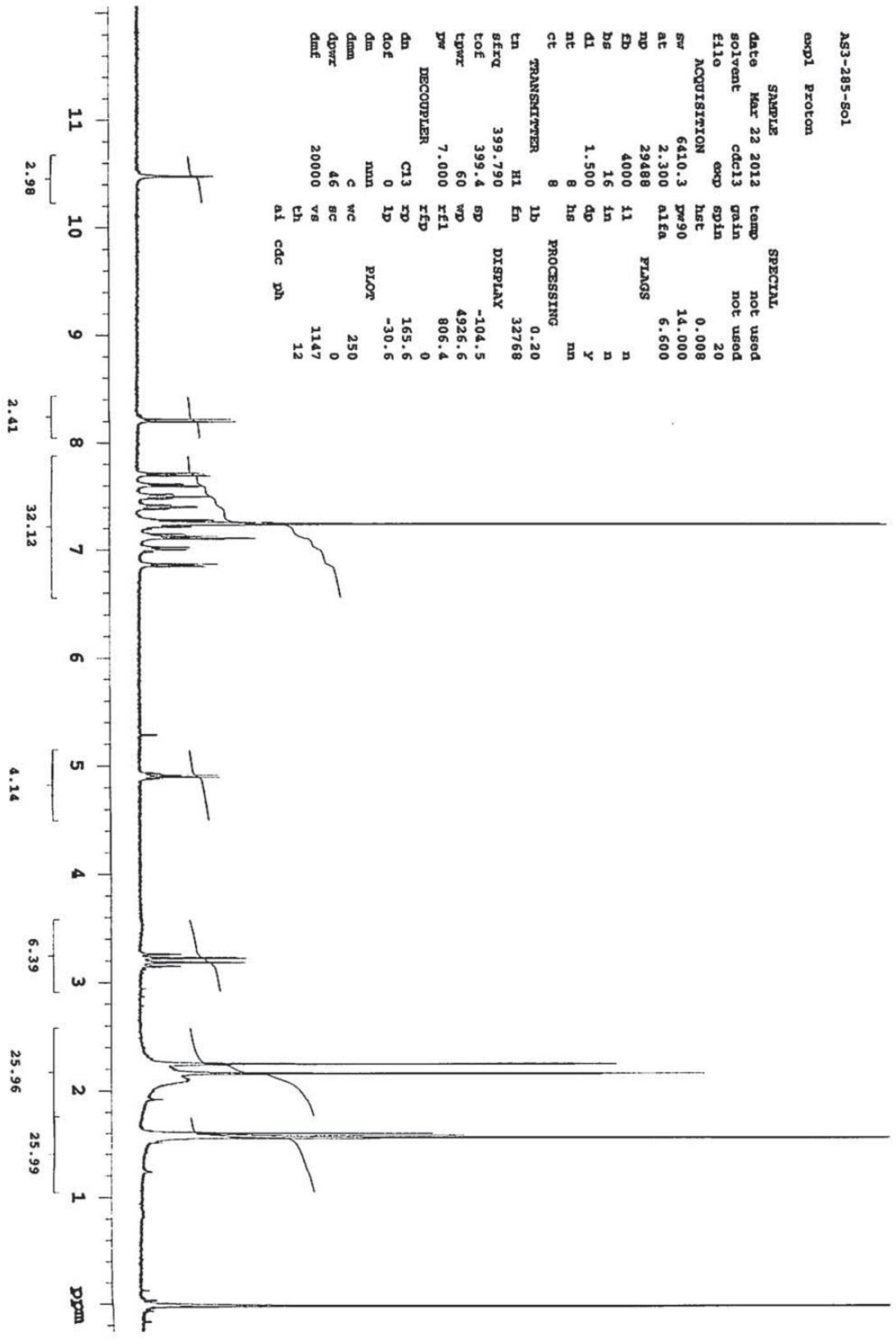

^{1}H NMR of (*R*)-**14b**

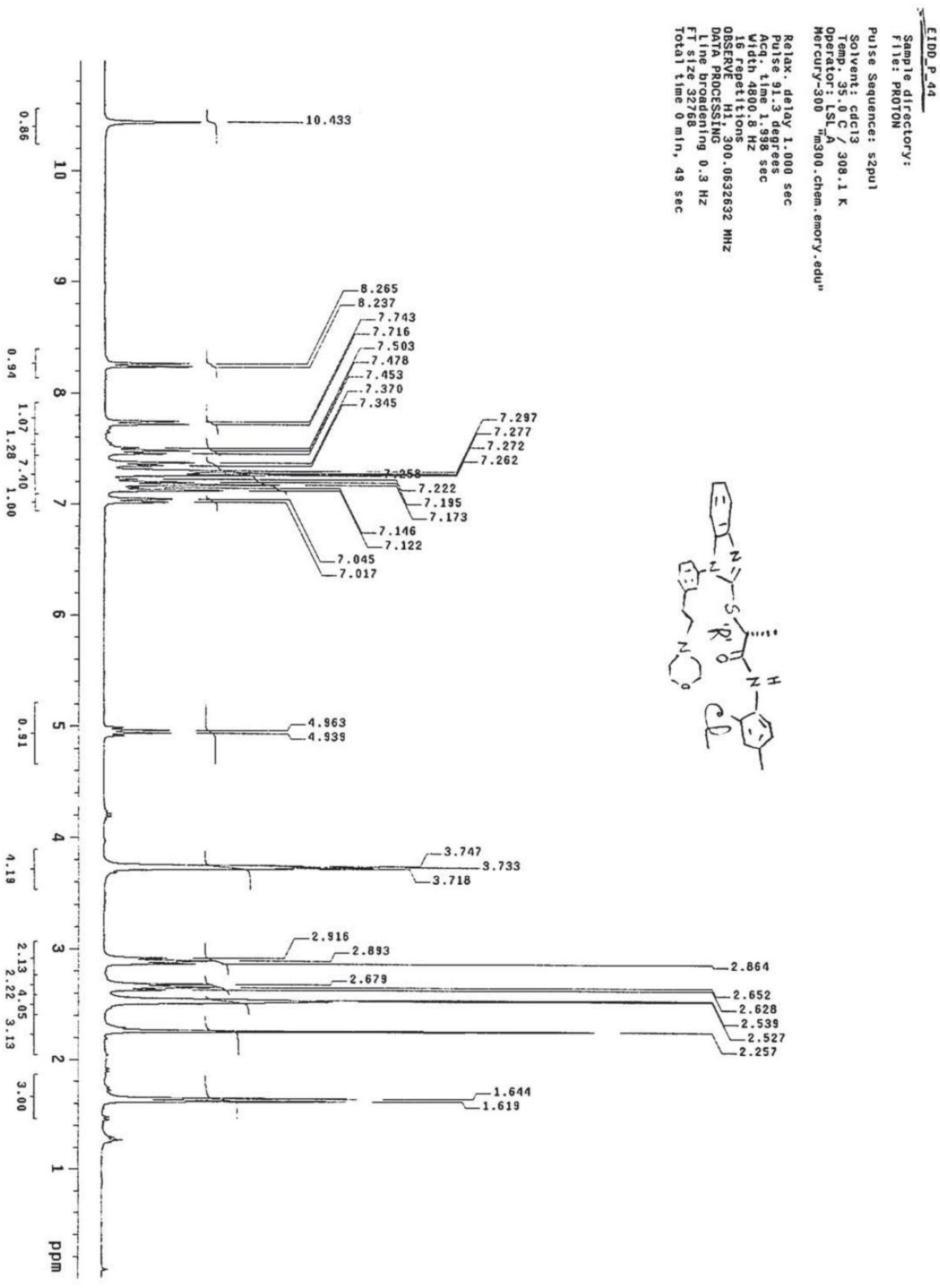

^{1}H NMR of (*S*)-**14b**

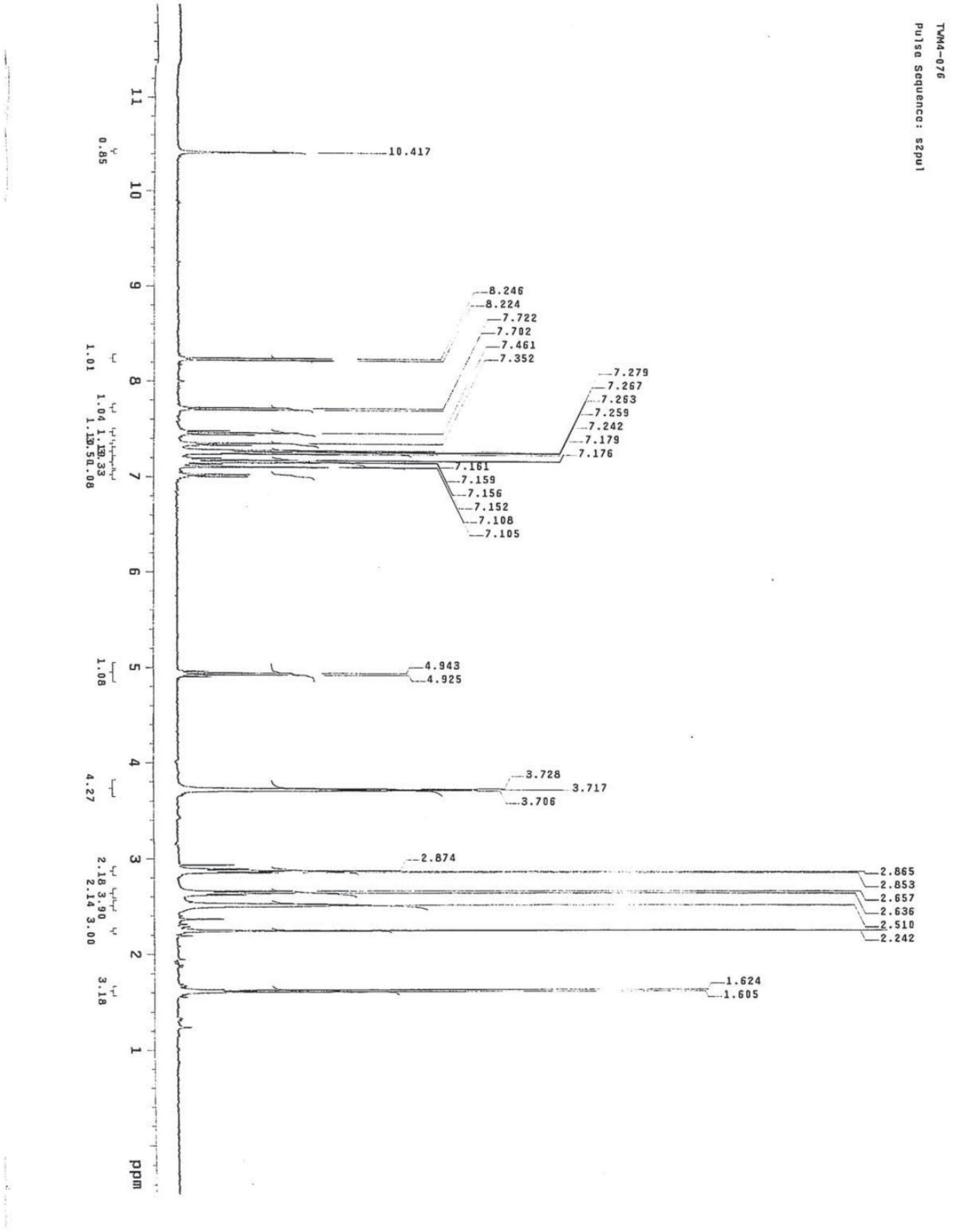

^{1}H NMR of (*R*)-**14c**

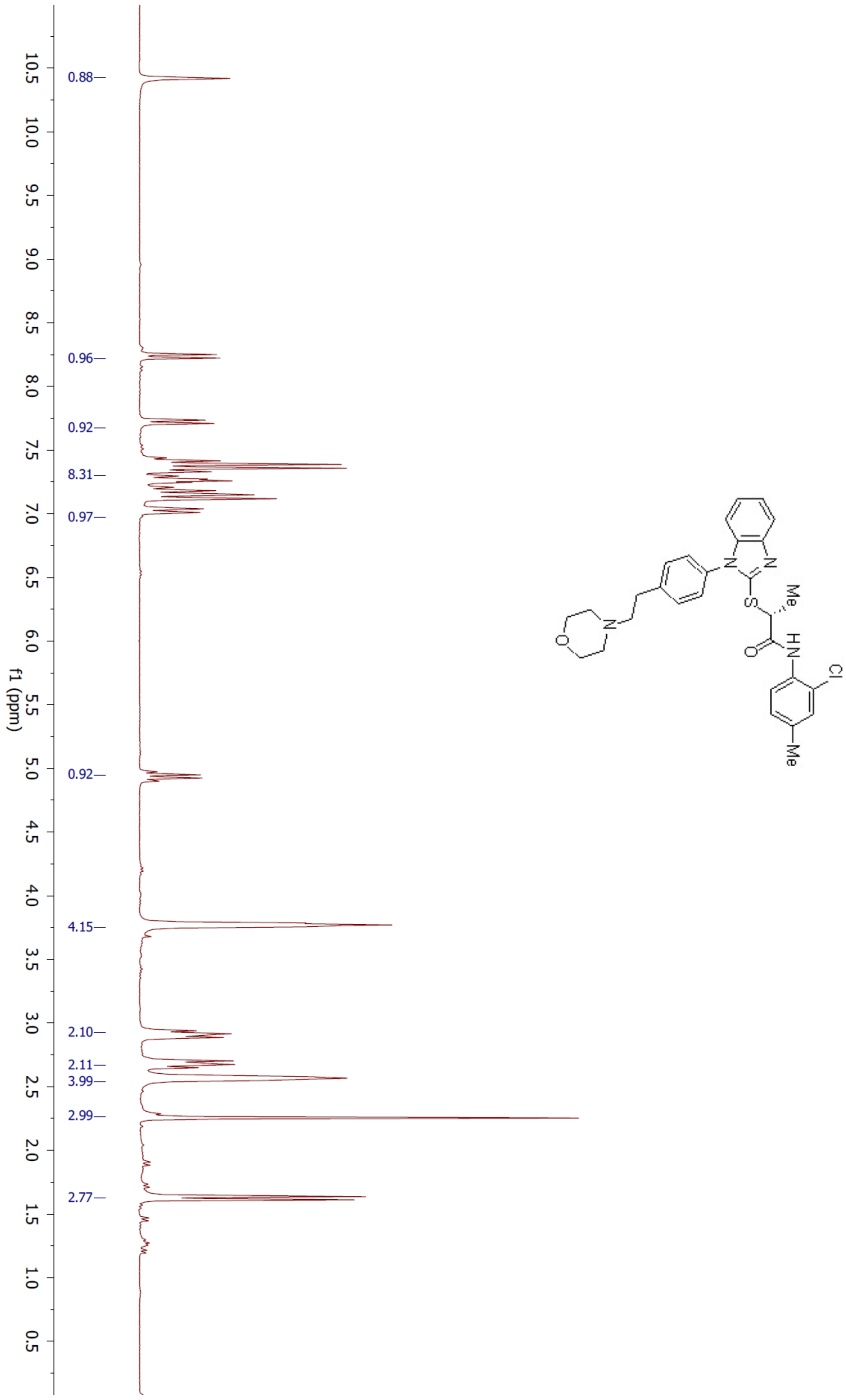

^{1}H NMR of (*S*)-**14c**

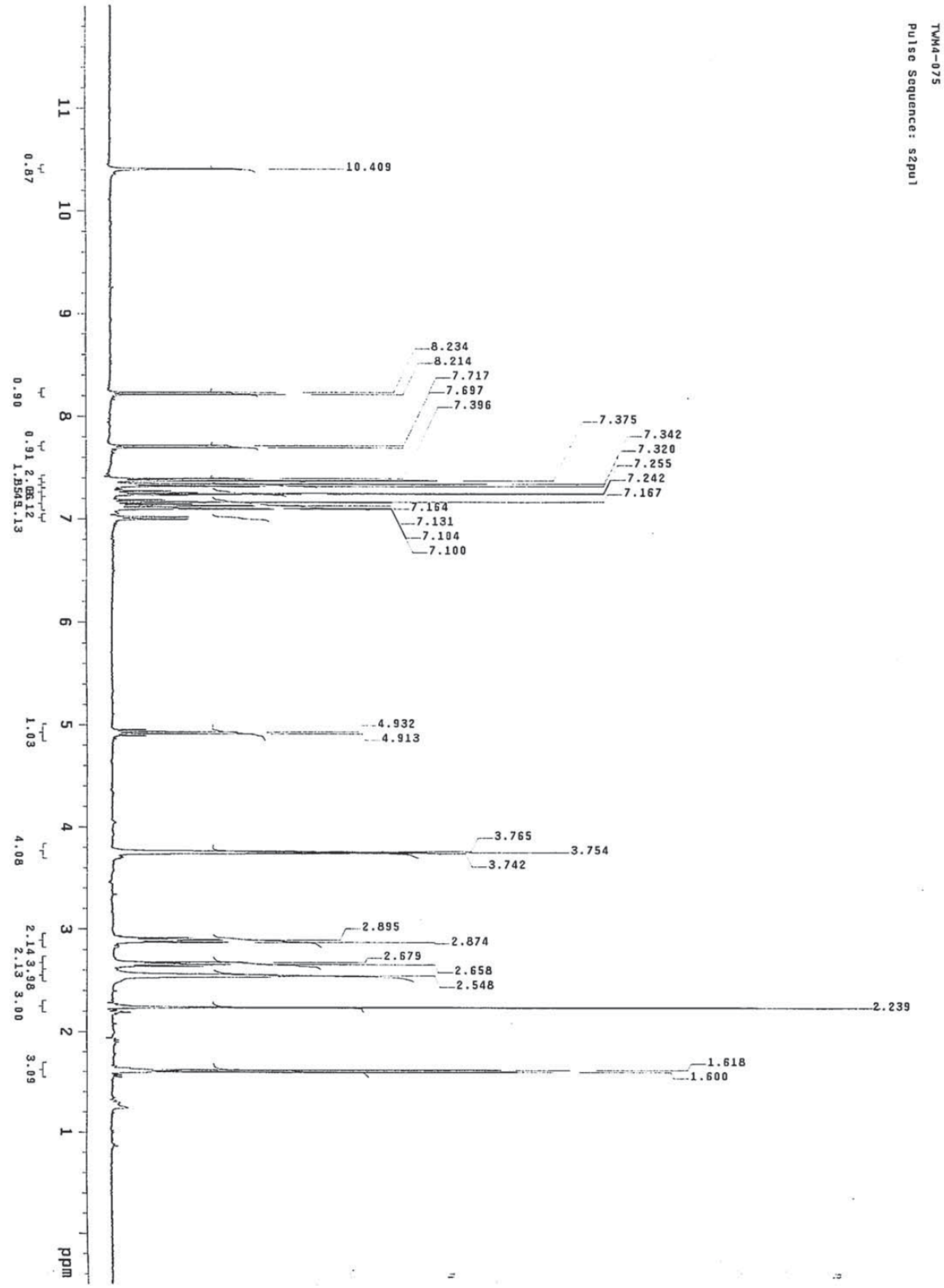

^{1}H NMR of Boc-**18a**

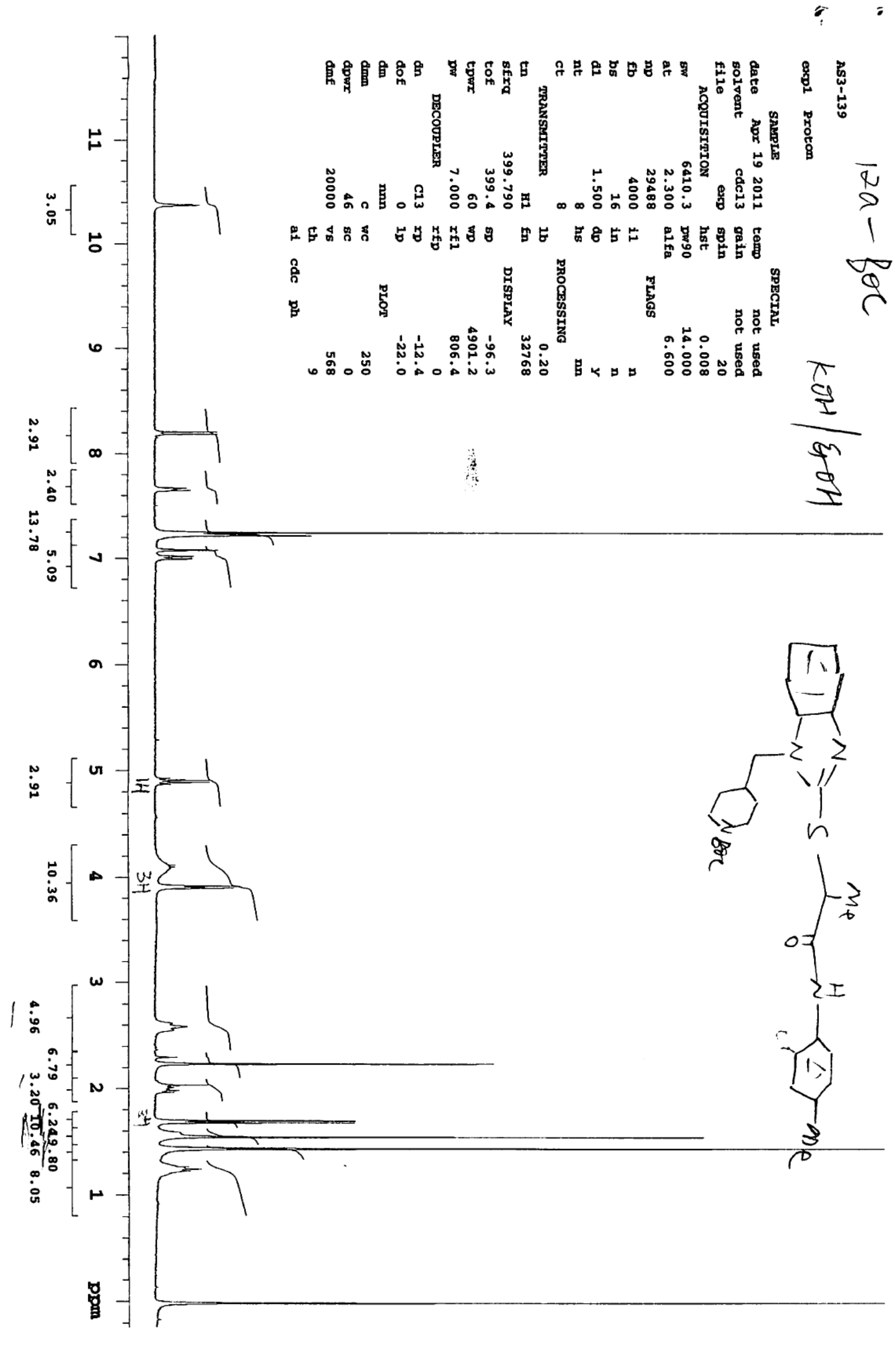

^{1}H NMR of **18a**

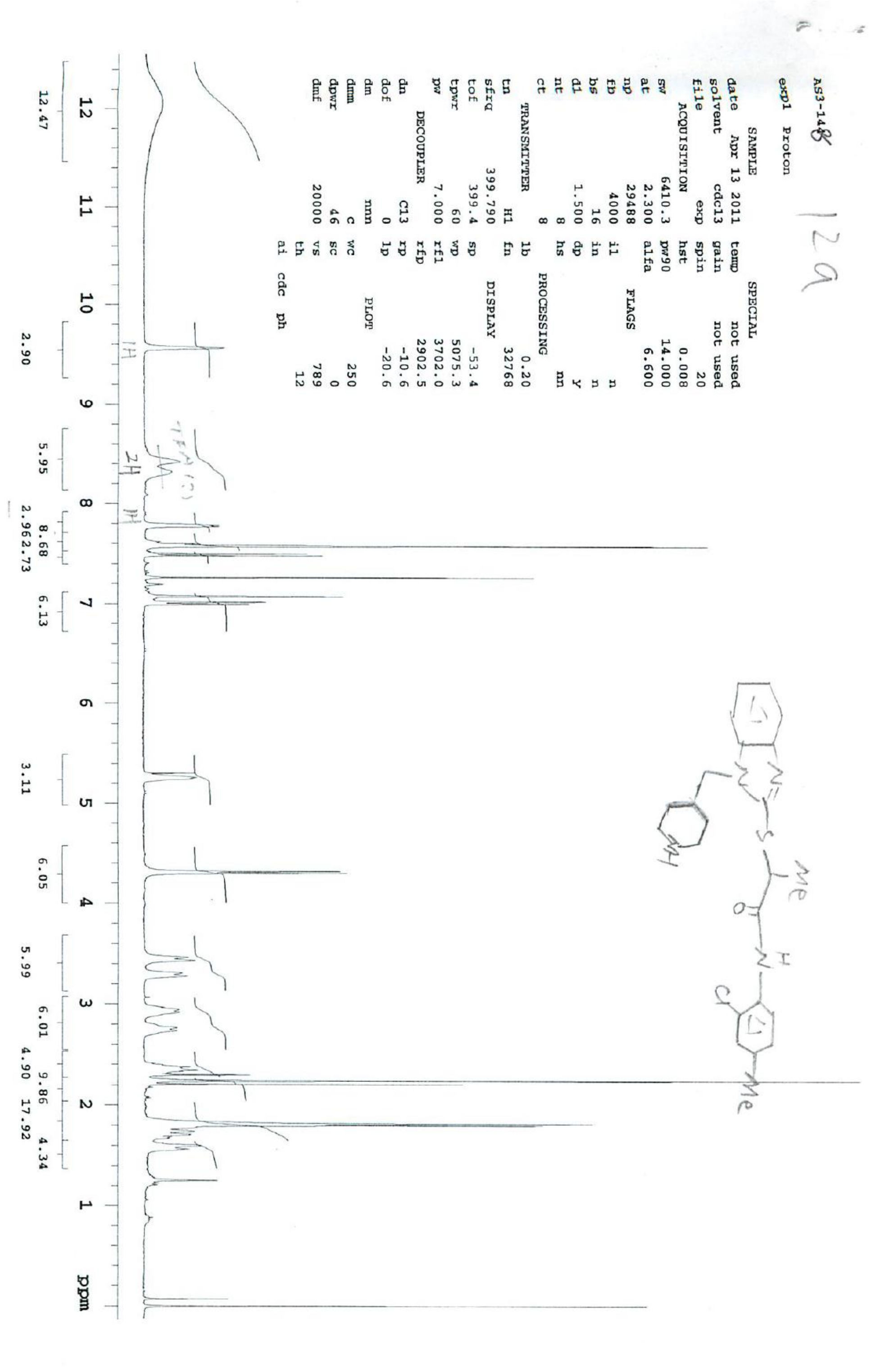

^{1}H NMR of **18b**

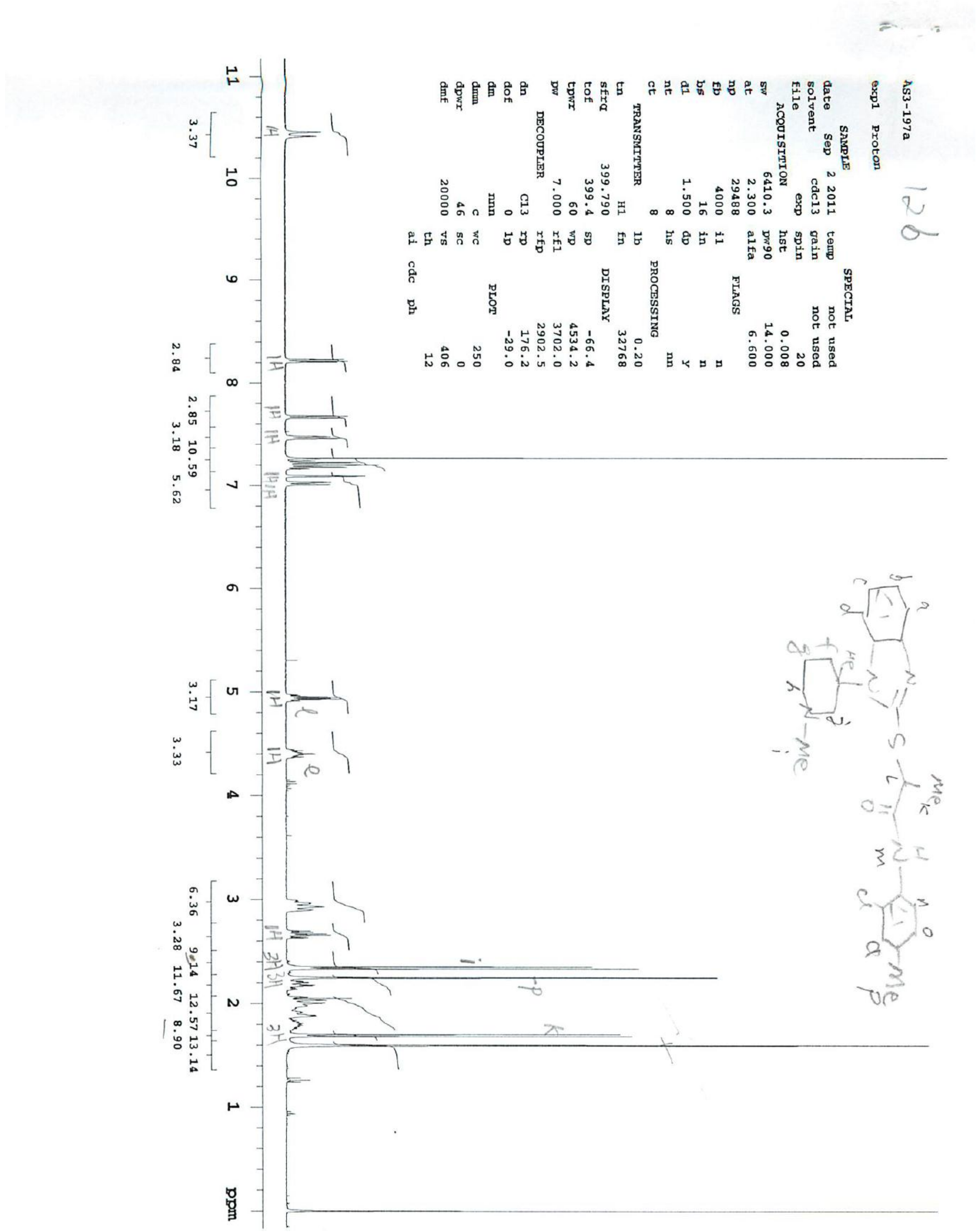

^{1}H NMR of **18c**

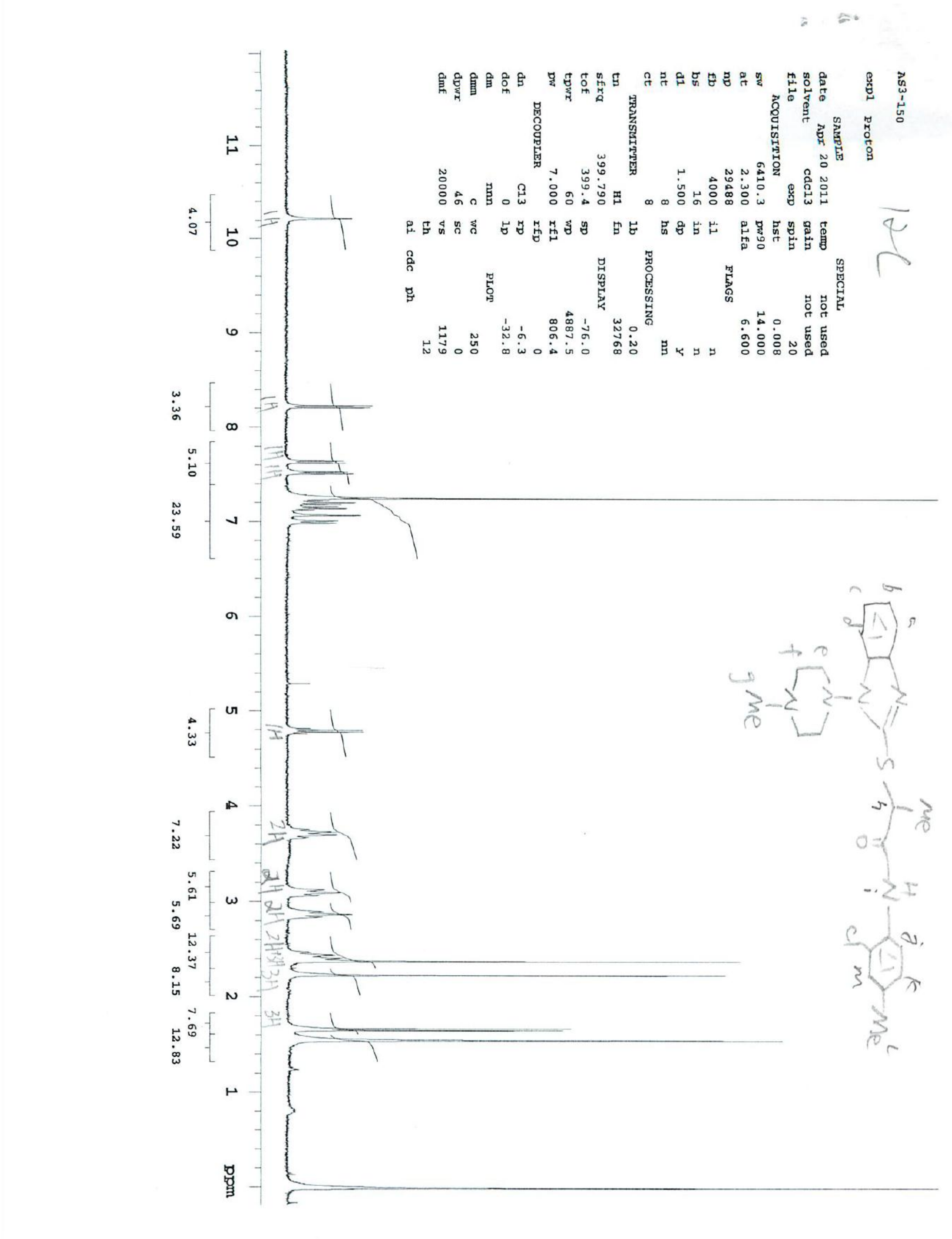

^{1}H NMR of **18d**

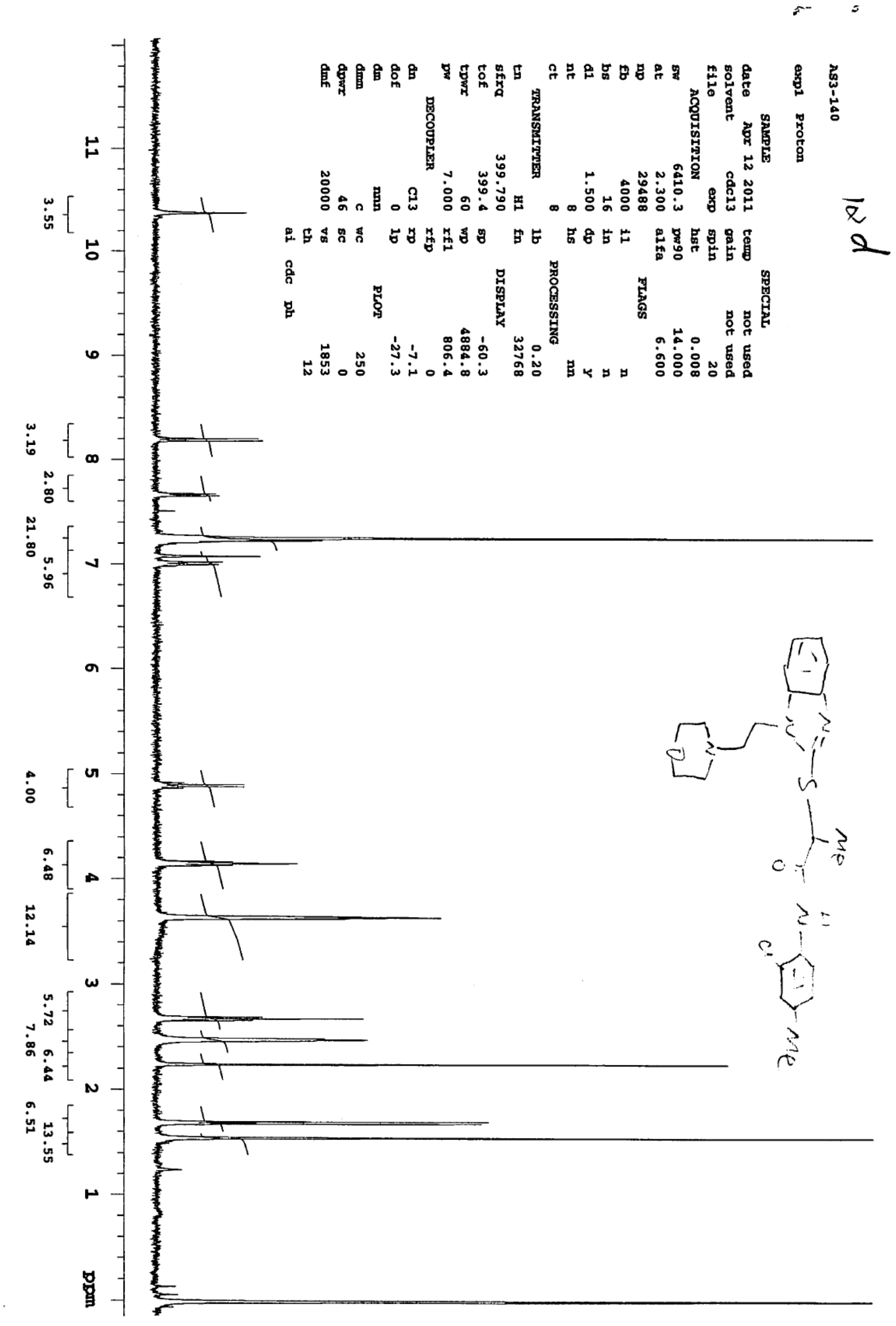

^{1}H NMR of **18e**

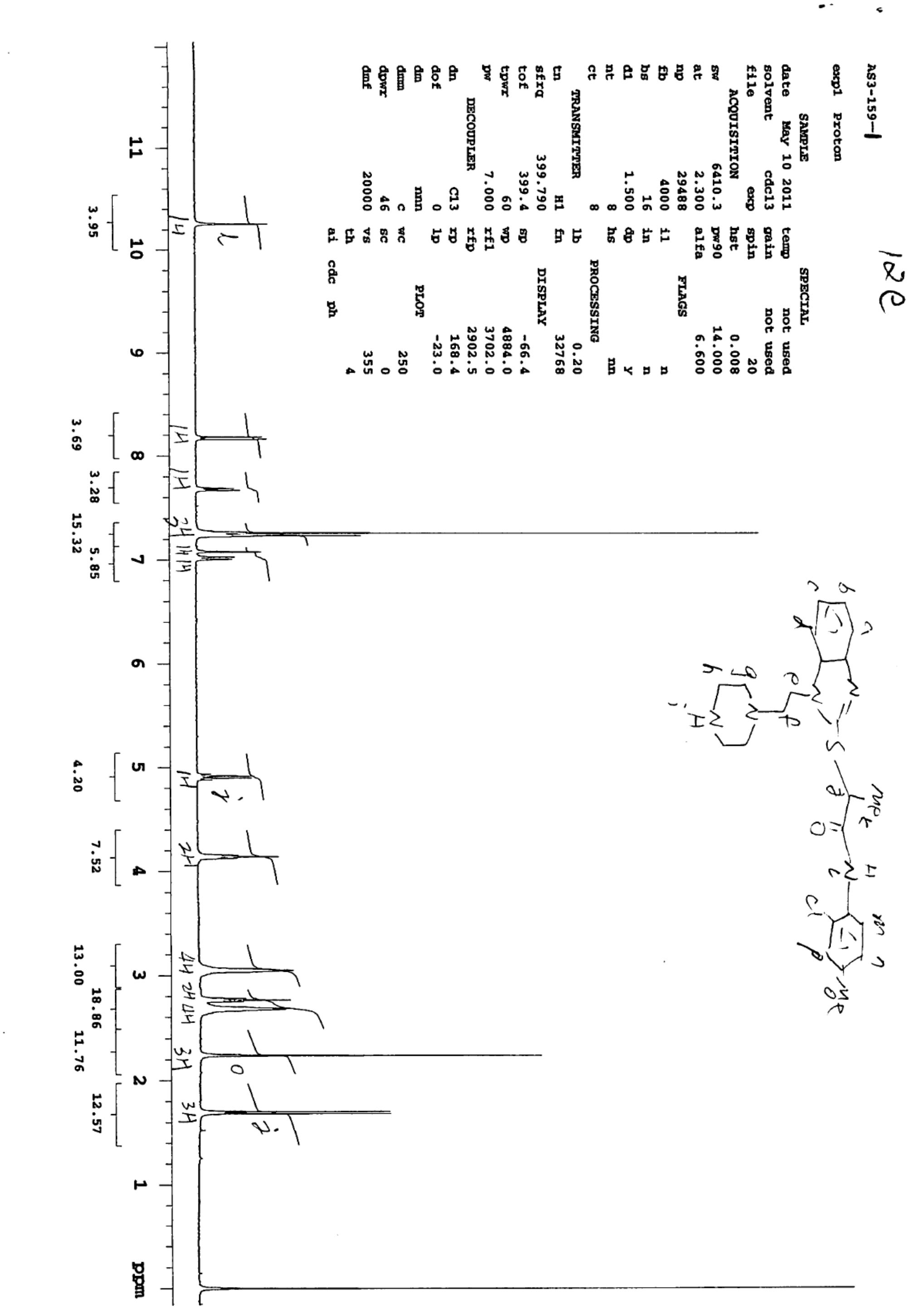

^{1}H NMR of **18f**

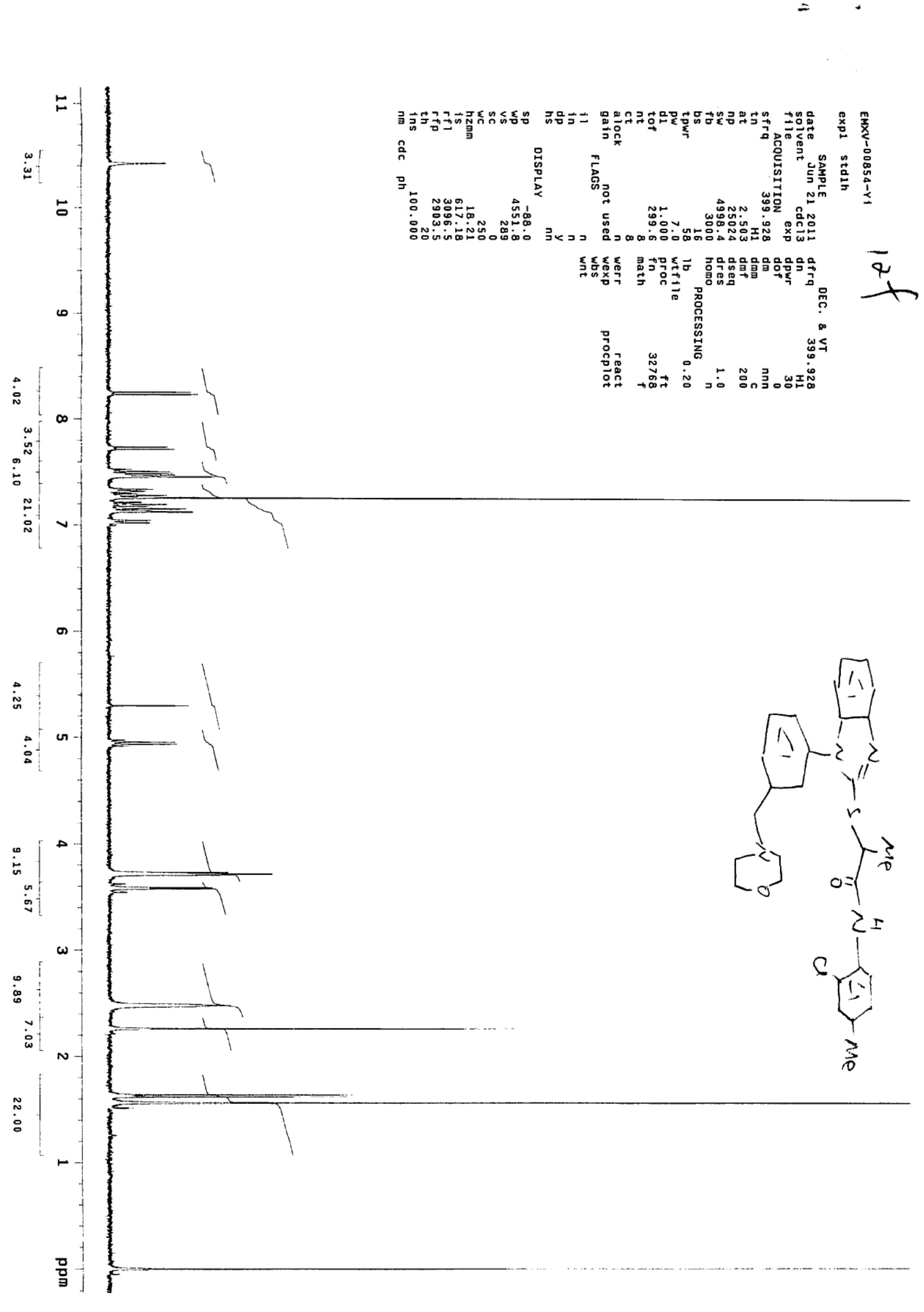

^{1}H NMR of **18g**

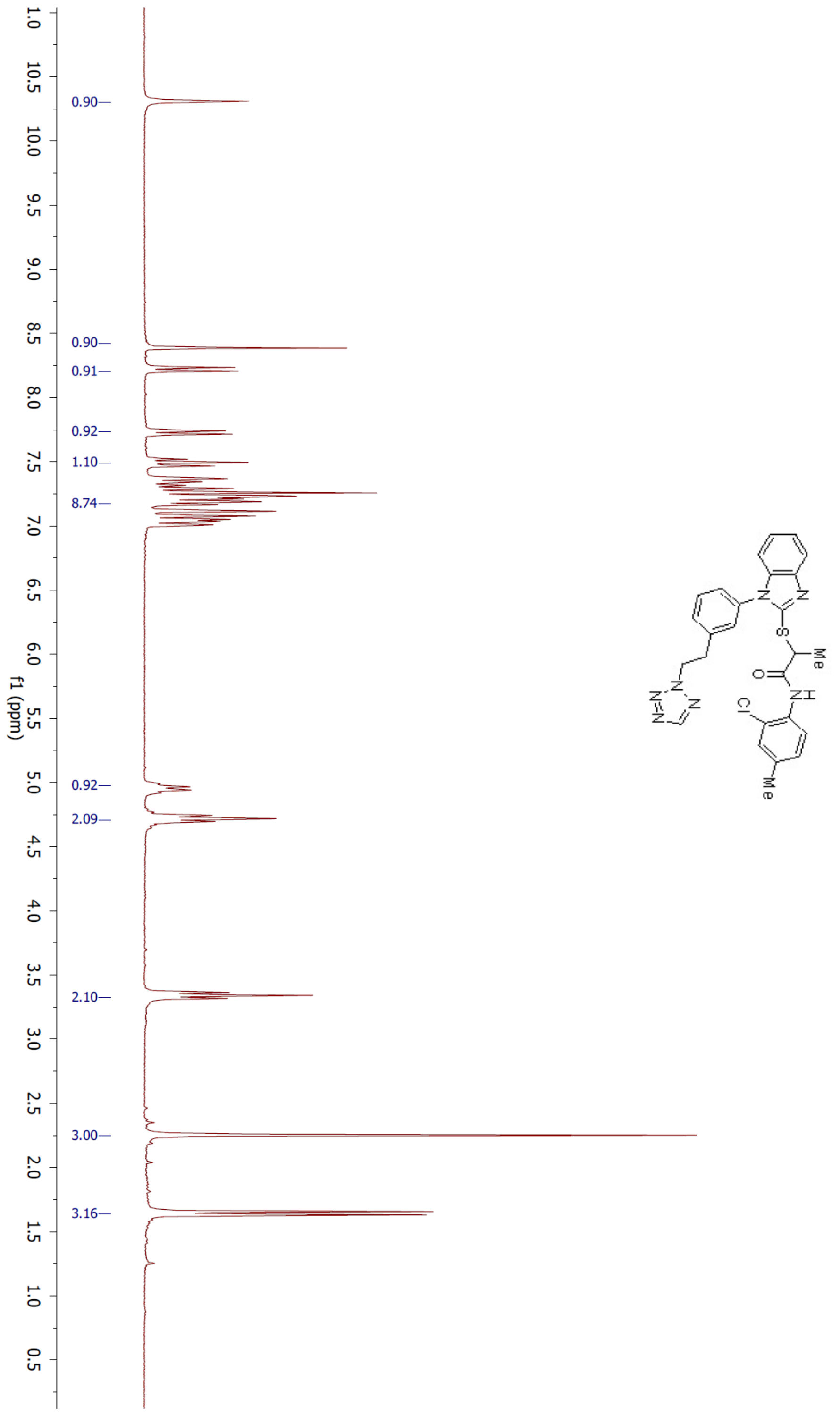

Reference

1. Sun, A.; Ndungu, J. M.; Krumm, S. A.; Yoon, Jeong-J.; Thepchatri, P.; Natchus, M.; Plemper, R. K.; Snyder, J. P. *ACS Med. Chem. Lett.* **2011,** *2,* 798-803.
